# Racial and ethnic differences in the effects of state firearm laws: a systematic review subgroup analysis

**DOI:** 10.1186/s40621-023-00477-y

**Published:** 2023-12-14

**Authors:** Rosanna Smart, Dionne Barnes-Proby, Pierrce Holmes, Terry L. Schell, Andrew R. Morral

**Affiliations:** 1 RAND Corporation, 1776 Main Street, PO Box 2138, Santa Monica, CA 90407-2138 USA; 2 RAND Corporation, Boston, MA USA; 3 RAND Corporation, Arlington, VA USA

**Keywords:** Violence, Firearm policy, Racial disparities

## Abstract

**Background:**

Despite growing evidence about how state-level firearm regulations affect overall rates of injury and death, little is known about whether potential harms or benefits of firearm laws are evenly distributed across demographic subgroups. In this systematic review, we synthesized available evidence on the extent to which firearm policies produce differential effects by race and ethnicity on injury, recreational or defensive gun use, and gun ownership or purchasing behaviors.

**Main body:**

We searched 13 databases for English-language studies published between 1995 and February 28, 2023 that estimated a relationship between firearm policy in the USA and one of eight outcomes, included a comparison group, evaluated time series data, and provided estimated policy effects differentiated by race or ethnicity. We used pre-specified criteria to evaluate the quality of inference and causal effect identification. By policy and outcome, we compared policy effects across studies and across racial/ethnic groups using two different ways to express effect sizes: incidence rate ratios (IRRs) and rate differences. Of 182 studies that used quasi-experimental methods to evaluate firearm policy effects, only 15 estimated policy effects differentiated by race or ethnicity. These 15 eligible studies provided 57 separate policy effect comparisons across race/ethnicity, 51 of which evaluated interpersonal violence. In IRR terms, there was little consistent evidence that policies produced significantly different effects for different racial/ethnic groups. However, because of different baseline homicide rates, similar relative effects for some policies (e.g., universal background checks) translated into significantly greater absolute differences in homicide rates among Black compared to white victims.

**Conclusions:**

The current literature does not support strong conclusions about whether state firearm policies differentially benefit or harm particular racial/ethnic groups. This largely reflects limited attention to these questions in the literature and challenges with detecting such effects given existing data availability and statistical power. Findings also emphasize the need for additional rigorous research that adopts a more explicit focus on testing for racial differences in firearm policy effects and that assesses the quality of race/ethnicity information in firearm injury and crime datasets.

**Supplementary Information:**

The online version contains supplementary material available at 10.1186/s40621-023-00477-y.

## Background

In 2021, nearly 49,000 people in the USA were killed by firearms, a 23% increase from just 2 years prior (Multiple Cause of Death by Single Race 2018–2021 on CDC WONDER Online Database 2022). While more than half of these deaths were suicides, homicides have driven rising rates of firearm mortality. Firearm homicide rates diverged sharply from non-firearm homicide rates starting in 2014 (Smart et al. 2022), beginning an increasing trend that was exacerbated during the COVID-19 pandemic (Kegler et al. 2022; Simon et al. 2022). Recent trends have exacerbated longstanding racial disparities in firearm homicide rates. In 2018, non-Hispanic Black people had firearm homicide rates 11 times higher than non-Hispanic white people (19.4 per 100,000 vs 1.7 per 100,000); in 2021, this risk ratio had increased to 15 (30.4 per 100,000 vs 2.1 per 100,000) (Multiple Cause of Death by Single Race 2018–2021 on CDC WONDER Online Database 2022). These disparities are tied to structural racism and inequities in education, employment, housing, and treatment by the criminal justice system, and addressing them may require a macro-level examination of how laws, institutions, and social systems influence firearm injury and death overall as well as across communities and populations (Bailey et al. 2021; Betz et al. 2021).

Firearm policies, many of which themselves have historical underpinnings rooted in efforts to deny Black people equal access to firearms (Cramer 1994; Winkler 2021), may play an important role in shaping racial and ethnic disparities in violent victimization and premature mortality (Sehgal 2020; Jahn et al. 2023; Tilstra et al. 2022). Growing evidence supports that some restrictive gun laws reduce total and firearm-specific homicide rates (Smart et al. 2023), and that some permissive gun laws, such as stand-your-ground laws, increase firearm deaths and homicides (Smart et al. 2023; Doucette et al. 2022; Yakubovich et al. 2021). However, whether these harms and benefits of gun laws are evenly distributed across demographic subgroups is not yet known. Firearm regulations that are effective in reducing rates of firearm violence may disproportionately benefit Black, Hispanic, and Native American populations given their higher rates of firearm homicide victimization (Smart et al. 2022; Kegler et al. 2022; Simon et al. 2022; Lawrence et al. 2023). Alternatively, given that non-White populations are substantially less likely to own or purchase firearms (Hemenway and Zhang 2022; Studdert et al. 2020), policies focused on firearms may produce larger effects among non-Hispanic white populations. The systems and institutions involved in the implementation and enforcement of these laws also likely matter; for example, firearm regulations that involve engagement with the criminal justice system or that impose restrictions based on criminal records may propagate longstanding racial inequalities in treatment by the criminal justice system. As firearm ownership, firearm violence, and firearm law enforcement each show strong racial differences, the extent to which gun policies produce differential effects by race and ethnicity may be expected to vary depending on policies’ mechanisms of action. Thus, a comprehensive understanding of the consequences of firearm regulations should include an assessment of potential racial or ethnic disparities in policy effects across a range of firearm policy classes.

To better understand the extent to which gun policies are associated with differential effects by race and ethnicity, this study conducts a subgroup analysis of a broader systematic review (Smart et al. in progress) evaluating the effects of 18 classes of firearm policy on eight outcomes of interest to stakeholders involved in firearm policy decisions. We aim to summarize available evidence from studies using causal inference methods to evaluate potential differential effects by race and ethnicity of state firearm laws on firearm injury and death as well as use of guns for self-protection or recreation.

## Methods

We performed this study in four stages. First, we updated our prior systematic review on the association between 18 classes of gun policy and eight outcomes (Smart et al. 2023) to include more recently published literature. Second, we conducted full text review of all studies that met our inclusion criteria for the broader review (i.e., used analytic designs that included a control group or comparison group and evaluated time series data with information both before and after the policy transition or transitions) to identify the subset of those studies that investigated policy effects for subpopulations defined by race or ethnicity. Third, we extracted policy effect estimates and inferential statistics specific to each subpopulation. Using information within each study, we derived estimates of subpopulation-specific policy effects in terms of incidence rate ratios (IRRs) and in terms of differences in incident rates. Finally, we assessed whether policy effects significantly differed from each other across racial and ethnic groups.

### Search, inclusion criteria, and data extraction

Our review follows Preferred Reporting Items for Systematic Reviews and Meta-Analyses guidelines. Identification of studies to inform these analyses followed from our ongoing systematic review of the effects of gun policy (Smart et al. 2023; Smart et al., in progress). The review protocol was registered in PROSPERO (CRD42019120105), and the protocol for this subgroup analysis was pre-registered via OSF.io (https://osf.io/ayzds). Search strategy, inclusion criteria, quality assessment, and effect coding were set a priori according to this protocol.

#### Search

In March 2023, we searched 13 databases (PubMed, PsycInfo, Index to Legal Periodicals, Social Science Abstracts, Web of Science, Criminal Justice Abstracts, National Criminal Justice Reference Service, Sociological Abstracts, EconLit, Business Source Complete, WorldCat, Scopus, and LawReviews [LexisNexis]) for English-language working papers, books, or peer-reviewed journal articles that estimated a relationship between one of 18 classes of gun policies and one of eight outcomes: violent crime, suicide, unintentional injury, defensive gun use, recreational gun use, gun industry outcomes, mass shootings, and police shootings. We used a broad set of search terms relevant for firearm policy (e.g., “gun,” “firearm,” “concealed carry”) and for outcomes (e.g., “suicide,” “murder,” “defensive gun use”; details in Additional file 1: Appendix A and B). The search timeframe, which covered August 1, 2020 to February 28, 2023, represented an update to our prior review search timeframe which covered studies published between January 1, 1995 through October 20, 2020.

#### Screening

Two trained reviewers independently screened titles and abstracts of identified articles, using a set of screening criteria developed by the research team. At the title and abstract screening stage, we only excluded studies if they did not relate to a firearm policy in the US context, assess one of the eight outcomes of interest, or include quantitative analyses. Discrepancies were resolved by consensus with input from a third reviewer as needed. Final inclusion of studies based on the full eligibility criteria (see below) was based on full-text evaluation. All screening was conducted in DistillerSR.

#### Inclusion criteria

Eligible studies were those that estimated an effect of one of 18 classes of gun policy on one of eight outcomes, evaluated time series data to establish that policies preceded their effects, and included a control or comparison group that was not exposed to the policy. For the purposes of this subgroup study, we additionally required that the article provided information on policy effects differentiated for subpopulations defined by race or ethnicity.

#### Extraction

Extracted information included metadata (e.g., title, authors); study features (e.g., timeframe, datasets); statistical methods (e.g., model type, analytic unit); population restrictions (e.g., white non-Hispanic individuals, black non-Hispanic individuals); and estimated effects (e.g., coefficient estimates, standard errors). One reviewer extracted data into a pretested standardized spreadsheet-based form. A second reviewer independently checked fields for accuracy; discrepancies were resolved by consensus.

We define effect comparisons at the study-policy-outcome level; for example, a study that conducted stratified analyses for how concealed carry laws and stand-your-ground laws affect homicides among white versus non-white populations would contribute two effect comparisons (one study, two policies, one outcome). Many studies provided multiple effect comparisons because they examined multiple different policies (e.g., waiting period laws and background check laws) or multiple different outcomes (e.g., homicides, firearm homicides, suicides, firearm suicides). When a study provided the required information for multiple different policies and/or outcomes, we extracted estimates for each; thus, a single study could contribute multiple comparisons.

However, many studies provide multiple effect estimates from several analyses of the same policy and outcome, for example, because the study estimated effects using different model specifications (e.g., with vs without time-varying covariates). When a study provided results for sub-populations of racial/ethnic groups, we extracted effect comparisons for the most representative population provided; for example, for a study that examined policy effects on black and white populations overall and by sex, we extracted the overall estimates only. If a study presented different estimates based on different model specifications, we extracted effect comparisons only from the specification subject to the fewest methodological concerns as based on our quality assessment criteria (see next section). This was typically the authors’ preferred specification unless otherwise noted.

#### Quality assessment

We assessed risk of bias using prespecified criteria shown to be important methodological considerations in quasi-experimental policy evaluations, and particularly in firearm policy research. While our quality assessment tool drew from criteria considered in the ROBINS-I (“Risk Of Bias In Non-randomized Studies-of Interventions”) tool (Sterne et al. 2016), we clarified or combined some of these criteria to better suit our context of firearm policy evaluations and attempt to address issues with evaluator burden and low interrater reliability that have been noted previously with practical implementation of ROBINS-I (Jeyaraman et al. 2020; Minozzi et al. 2020). Details on these decisions are in Additional file 1: Appendix C.

Quality assessment for each effect comparison (study-outcome-policy) was conducted through discussion with the full review team regarding the following domains:

- threats to causal identification (e.g., failure to adjust for confounds)
- number of treated units, control units, and pre- and post-treatment data
- policy classification
- outcome missingness
- evidence of model overfit
- validity of statistical assumptions and inferential statistics
- other (e.g., sensitivity of results to model specification)

We indicated whether each effect comparison had an issue on each domain as described in Additional file 1: Appendix C. Quality criteria were used to narratively describe the quality of the underlying studies contributing to the review.

#### Effect size calculations

For each effect and sub-population, we code policy effects in two ways, as incidence rate ratios (IRRs) and as rate differences, both of which are plausibly of interest in assessing the role of firearm policies in increasing or reducing racial and ethnic differences in outcomes. Nearly all studies that met our inclusion criteria used model specifications whereby policy effects were expressed as relative rate ratios (e.g., as IRRs). Comparing IRRs across racial/ethnic groups will provide an indication of whether policies produced greater relative benefits or harms for different groups. However, for many of our outcomes of interest (e.g., firearm homicides), baseline rates vary substantially by race and ethnicity. Given different baseline rates, policies that produce the same relative effects for different populations (i.e., policies that have an effect that is the same in IRR terms for all population subgroups) will have substantively different implications for the number of additional deaths or lives saved. To highlight this, in addition to presenting effects in IRR terms, we also present effect estimates (and their associated 95% confidence intervals [CIs]) as differences in per capita rates using the calculations described in Additional file 1: Appendix D.

We also seek to characterize whether policy effects for each racial/ethnic group significantly differ from each other, e.g., whether the policy has significantly larger effects for one racial group versus another. For studies that present the relevant statistics for this hypothesis test, we use the information provided in the study. For studies that do not provide this information (e.g., a study only conducts analyses stratified by race, with no additional analyses), we use the information within the study to calculate the relevant *p* value for differences by race/ethnicity under the assumption that the estimates for each racial/ethnic group can be treated as being drawn from independent samples. As before, we consider significance of differences in policy effects by race/ethnicity in both relative (e.g., do policies reduce firearm death rates for black and nonblack populations by the same proportion) and in absolute terms (e.g., do policies reduce firearm death rates for black and nonblack populations by the same amount). We refer to results from these tests as differential effects on rate ratios or as differential effects on rate differences (see Additional file 1: Appendix D for calculations).

#### Synthesis of results

For each policy and outcome, we narratively summarize conclusions of evidence based on combined assessment of the number of studies and their methodological quality, effect magnitudes and uncertainty of the effects, and directional consistency of effects across studies. When multiple estimates for a given policy and outcome are available, we do not calculate meta-analytic estimates of policy effects, because nearly all studies use the same or similar data, meaning effect estimates cannot be treated as independent. However, we visualize the evidence using forest plot figures to show estimates from studies without serious methodological weaknesses, using separate figures to present effects expressed as mortality rate ratios and as mortality rate differences.

## Results

**Fig. 1:**
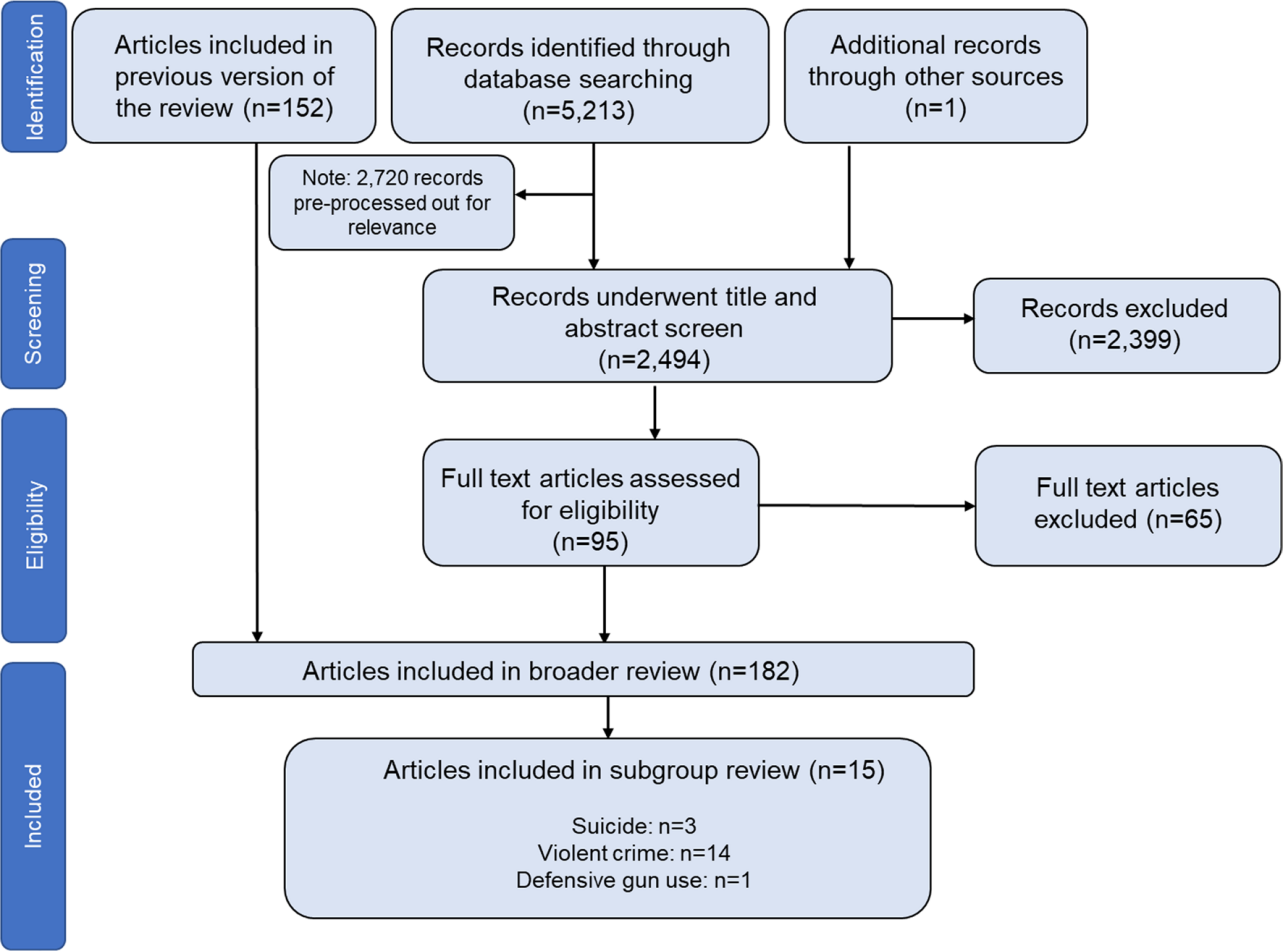
Study flow diagram of literature search and selection of studies. *Notes*: The one study identified outside of the search was a peer-reviewed published version of an included working paper that was inadvertently screened out as a duplicate in a prior edition of the review. The peer-reviewed publication now supersedes the working paper

Our updated search resulted in 5214 unique records, of which 2720 records were pre-processed out for lack of relevance (Fig. 1). Thus, a total of 2494 records underwent title and abstract screen as part of the review update. From those, 95 merited full text review, of which 30 merited inclusion in our larger systematic review. Combined with the 152 included articles from Smart et al. (in progress), this resulted in 182 total articles that were screened further for eligibility for this subgroup analysis. Of these 182 articles, 15 met our inclusion criteria of investigating differential policy effects by race or ethnicity.

In total, the 15 included studies provided 57 usable effect comparisons to inform potential differences by race or ethnicity in the impacts of state gun policies (Table 1). Most studies evaluated effects for homicide, violent crime, or nonfatal assaults (14 studies; 51 effect comparisons), with only three studies (five comparisons) evaluating differential effects on suicide or self-injury outcomes, and one study (one comparisons) evaluating defensive gun use outcomes.

**Table 1:** Characteristics of included studies

Study	Setting (analytic unit)	Model link function	Period	Racial or ethnic groups	Outcome	Policies evaluated
Anderson et al. (2021)	USA (State)	Log-linear	1985–2013	Under age 18: White, Nonwhite	1. Firearm murder	1. Child access prevention law
D’Alessio et al. (2022)	Multi-State (City)	Linear	2002–2015	Black, Nonblack	1. Firearm crime (as proportion of all crime)	1. Stand-your-ground
Dalafave (2020)	USA (State)	Log-linear	1990–2018	White, Nonwhite	1. Homicide 2. Firearm homicide 3. Non-firearm homicide 4. Suicide 5. Firearm suicide 6. Non-firearm suicide	1. Extreme risk protection orders
Degli Esposti et al. (2022)	USA (State)	Quasi-Poisson	1999–2017	White, Nonwhite	1. Homicide 2. Firearm homicide	1. Stand-your-ground
Edwards et al. (2018)	USA (State)	Log-linear	1990–2013	White, Nonwhite	1. Firearm suicide	1. Waiting period
Hepburn et al. (2004)	USA (State)	Negative binomial	1979–1998	Men, ages 35+: White	1. Homicide	1. Shall-issue law 2. Background check requirement 3. Waiting period
Kaufman et al. (2020)	USA (State)	Poisson	1999–2017	Non-Hispanic Black, Non-Hispanic White	1. Firearm homicide	1. Background check requirement
Knopov et al. (2019)	USA (State)	Log-linear	1991–2016	White, Black	1. Homicide	1. Background check requirement 2. Concealed carry laws 3. Permit-to-purchase licensing 4. Stand-your-ground law 5. DV prohibitions and surrender
Lott and Mustard (1997)	USA (County)	Log-linear	1977–1992	White, Black, Hispanic	1. Murder	1. Shall-issue law
McClellan and Tekin (2017)	USA (State)	Poisson, Log-linear	2000–2010	White, Black	1. Firearm homicide 2. Firearm assault injury 3. Justifiable homicide	1. Stand-your-ground law
Olson and Maltz (2001)	USA (County)	Log-linear	1977–1992	White, Nonwhite	1. Murder 2. Firearm murder	1. Shall-issue law
Rubin and Dezhbakhsh (2003)	USA (County)	Linear	1982–1992	Black, Nonblack	1. Violent crime	1. Shall-issue law
Pear et al. (2022)	California (County)	Synthetic control	2005–2019	Non-Hispanic White, Black or Hispanic	1. Firearm assault injury 2. Intentional firearm self-injury	1. Extreme risk protection orders
Rochford et al. (2022)	Multi-State (State)	Negative binomial	2013–2017	Unmarried victims: White, Nonwhite	1. IPH 2. Firearm IPH	1. DV prohibitions
Wallin et al. (2022)	USA (State)	Negative binomial	1981–2013	White, Black	1. IPH 2. Firearm IPH	1. DV prohibitions and surrender 2. Background check requirement 3. Permit-to-purchase licensing

All studies evaluated the US context and used quasi-experimental designs that evaluated pre-post-policy data and included a control group without the policy or policies of interest (or with limited implementation of the policy of interest).

DV, domestic violence; IPH, intimate partner homicide

The most commonly studied policies were concealed carry laws (five studies; six effect comparisons), followed by stand-your-ground laws (four studies; nine comparisons), background check requirements (four studies; seven comparisons), and firearm prohibitions related to domestic violence (DV; three studies; 21 comparisons). Two studies (two effect comparisons) contributed to evaluating potential differential effects of waiting periods, two studies (three comparisons) contributed to evaluating differential effects of permit-to-purchase licensing, and two studies (five comparisons) assessed extreme risk protection order (ERPO) laws. One study (one comparisons) evaluated child access prevention laws.

Classification of racial and ethnic groups varied slightly across studies (Table 1). Nine studies grouped individuals as white or non-white, two studies grouped individuals as black or nonblack, and three looked at white individuals separately from black individuals but excluded other races from the analyses. Only three studies considered Hispanic ethnicity.

Most studies used a quasi-experimental differences-in-differences type design, controlling for year fixed-effects and geographic fixed- or random-effects. One study (Pear et al. 2022) used a synthetic control method approach, two studies used methods that controlled for state fixed effects but modeled time as a parametric function rather than as year fixed effects (Degli Esposti et al. 2022; Wallin et al. 2022), and one study used a regression-based approach that controlled for year but not geographic unit effects (Rochford et al. 2022). Differential policy effects by race/ethnicity were generally assessed based on models stratified by race/ethnicity (Anderson et al. 2021; Dalafave 2020; Degli Esposti et al. 2022; Edwards et al. 2018; Kaufman et al. 2020; McClellan and Tekin 2017; Pear et al. 2022; Rochford et al. 2022; Wallin et al. 2022); two studies directly estimated interaction terms (D’Alessio et al. 2022; Knopov et al. 2019); two early concealed carry law studies examined policy effects on the racial composition of murder victims (Lott and Mustard 1997; Olson and Maltz 2001); one study estimated the association of county-level racial composition with predicted changes in violent crime following shall-issue law adoption (Rubin and Dezhbakhsh 2003); and another concealed carry law study conducted a sub-analysis focused on the “high-exposure” group of white men aged 35 and older, who were claimed to comprise a large proportion of concealed carry permit holders (Hepburn et al. 2004). Only six studies (contributing 12 of the 57 comparisons) included results from statistical tests for racial or ethnic differences in policy effects (D’Alessio et al. 2022; Degli Esposti et al. 2022; Knopov et al. 2019; Lott and Mustard 1997; Olson and Maltz 2001; Rubin and Dezhbakhsh 2003).

### Policies regulating who may legally own, purchase, or possess firearms

Five studies (Dalafave 2020; Knopov et al. 2019; Pear et al. 2022; Rochford et al. 2022; Wallin et al. 2022) assessed differential effects of policies prohibiting the possession and purchase of firearms by certain types of individuals at risk of violence. Two studies (Knopov et al. 2019; Wallin et al. 2022) had only minor methodological concerns (Wallin et al. 2022); the others had serious (Dalafave 2020; Pear et al. 2022) or critical concerns (Rochford et al. 2022).

Wallin et al. (2022) used data from 1981 to 2013 to evaluate how firearm prohibitions related to DV affect intimate partner homicide (IPH), with a focus on understanding differences across white and black IPH victims. They considered a range of different types of DV prohibition policies, including whether state laws allow law enforcement to remove firearms at the scene of a DV incident. For white victims, they found that state domestic violence restraining order (DVRO) prohibitions were associated with significant reductions in total (IRR = 0.90; 95% CI = 0.83–0.98) and firearm-related intimate partner homicides (IRR = 0.89 95% CI = 0.80–0.99), particularly when accompanied with firearm surrender requirements. However, stalking misdemeanor prohibitions were associated with significant increases in IPH among white victims (IRR = 1.20, 95% CI = 1.04–1.38). Estimated effects for black victims were imprecise for all state laws evaluated. Effects were negative for the two federal laws evaluated, but only one of these met our inclusion criteria of having a comparison group: federal law prohibiting firearm possession among domestic violent misdemeanants, which had differential effects across states due to preexisting state variation in assault statutes, had a suggestive effect consistent with reducing firearm-related IPH among this population (IRR = 0.87; 95% CI = 0.75–1.01; *p* = 0.07). While the authors did not explicitly test whether estimates differed by race, treating both samples as independent shows only one policy as having suggestive differential effects by race in mortality rate ratio terms: state violent misdemeanor prohibitions lead to greater relative benefits for white victims of IPH (*p* = 0.06) and firearm IPH (*p* = 0.07). No policies showed differential effects by race in mortality rate difference terms.

This finding seems to contrast with results in Knopov et al. (2019), which instead examined all homicides (not only intimate partner homicides) from 1991 to 2016. Operationalizing the policy variable as prohibitions on handgun possession for those with violent misdemeanors or subject to DVRO (with surrender requirements), they instead found that these prohibitions produced larger benefits for black homicide victims (IRR = 0.87, 95% CI = 0.81–0.94), and these reductions were significantly larger than those for white homicide victims (IRR = 0.97, 95% CI = 0.92–1.02); differential effects by race were significant on both mortality rate ratios (*p* = 0.03) and on mortality rate differences (*p* = 0.001).

Rochford et al. (2022) instead assessed how state laws closing the “boyfriend loophole” influenced intimate partner homicides of unmarried individuals. Based on a short panel of data for 18 states from 2013 to 2017, they found that extending DVRO and/or DV misdemeanor firearm prohibitions to dating partners resulted in significantly lower rates of IPH only for unmarried white individuals, with IRRs ranging from 0.55 to 0.72 depending on the particular policy operationalization. Estimated policy effects on IPH among unmarried victims of color were instead large and positive, resulting in effects that significantly differed from those for white victims when policy effects were scaled as rate ratios (all *p* < 0.05) as well as when scaled as differences in rates (all *p* < 0.04). However, this study’s analysis controlled only for national trends and a limited set of time-varying state covariates, several of which are plausibly endogenous to the policies (e.g., ratio of firearm suicides to suicide, 2019 violent crime rate). This, combined with fewer than three states transitioning laws during the study period, leads to critical concerns with bias in the effect estimates, which should not be taken as causal and instead likely reflect differences in IPH rates by race that are correlated with states that do versus do not have firearm prohibition policies addressing dating partners.

Finally, two studies assessed the effects of ERPO laws on firearm violence and conducted analyses stratified by race. Pear et al. (2022) evaluated the effects of San Diego’s implementation of California’s ERPO law relative to other California counties that issued very few ERPOs. Combining hospitalization and mortality data from 2005 to 2019, their synthetic control method approach produced highly imprecise estimates of effects for ERPO implementation in San Diego on both intentional firearm self-harm injuries and on firearm assault injuries. Estimates for firearm assault injury stratified by Black or Hispanic versus non-Hispanic white were similar (IRRs of 0.76 and 0.72, respectively) but imprecise. Uncertainty around the race-specific estimates results in differential effects by race that are not significant as rate ratios (*p* = 0.99) or as rate differences (*p* = 0.36) Furthermore, there are serious concerns with the validity of the approaches’ identifying assumptions for estimating effects on firearm assault injury among Black and Hispanic individuals given very poor pre-period fit between San Diego and its synthetic control.

Comparing changes in state-level homicide and suicide rates after state ERPO adoption to changes in states without such laws, Dalafave (2020) found that ERPO laws significantly reduced state suicide rates for both white (IRR = 0.96; 95% CI = 0.94–0.99) and nonwhite (IRR = 0.87; 95% CI = 0.78–0.97) populations. Results suggested possibly larger reductions in suicide among nonwhite compared to white populations when evaluated as rate ratios (*p* = 0.08). However, given lower baseline suicide rates among nonwhite populations, these differences were not significant when converted to rate difference terms (*p* = 0.12). For homicide outcomes, Dalafave (2020) found no significant effects of ERPO laws overall and no significant differences by race (*p* values range from 0.16 to 0.59). However, given the analyses dropped state-year observations with fewer than 10 deaths (due to suppression in the outcome dataset), there are serious concerns with potential bias in this study’s estimates and inferential statistics.

### Policies regulating the sale and transfer of firearms

Five studies (Edwards et al. 2018; Hepburn et al. 2004; Kaufman et al. 2020; Knopov et al. 2019; Wallin et al. 2022) assessed whether effects of policies regulating firearm sales and transfers differ across racial/ethnic groups. Four studies evaluated background check laws (Hepburn et al. 2004; Kaufman et al. 2020; Knopov et al. 2019; Wallin et al. 2022); two separately estimated the effects of permit-to-purchase requirements (Knopov et al. 2019; Wallin et al. 2022); and two (Edwards et al. 2018; Hepburn et al. 2004) evaluated waiting period requirements. All had serious methodological concerns, with the exception of estimates from one study (Knopov et al. 2019) examining the effects of universal background check laws on homicides that had only minor concerns.

Using data from 1991 to 2016, Knopov et al. (2019) found significant reductions in age-adjusted homicide rates associated with universal background checks (IRR = 0.89; 95% CI = 0.81–0.97), with tests indicating no significant differences across white and black victims in rate ratio terms. However, because of racial differences in baseline homicide rates, similar relative effects translate into significantly greater reductions in absolute terms for non-Hispanic black relative to non-Hispanic white victims (*p* = 0.03). Estimated effects and inferential tests were nearly identical for permit-to-purchase requirements, although with only two states changing permit-to-purchase requirements in the study period, there are serious concerns with the reliability of these estimates and their inferential statistics.

In models stratified by non-Hispanic black versus non-Hispanic white victims, Kaufman et al. (2020) estimated that universal background check laws reduced firearm homicides among non-Hispanic black individuals (IRR = 0.81, 95% CI = 0.70–0.94) and had uncertain effects on firearm homicides of non-Hispanic white individuals (IRR = 0.93, 95% CI = 0.73–1.20). Estimates by race did not significantly differ from each in rate ratio terms (*p* = 0.35). However, because of racial differences in baseline firearm homicide rates, similar relative effects translate into significantly greater reductions in rate difference terms for non-Hispanic black relative to non-Hispanic white victims (*p* = 0.004). However, this study dropped individual state-years with suppressed data, introducing serious concerns with potential bias in estimated effects.

Using data from an earlier timeframe (1979 to 1998), Hepburn et al. (2004) found no significant effects of handgun background check requirements on homicide rates overall (IRR = 1.02; 95% CI = 0.93–1.12) or among white males ages 35 and older (IRR = 0.97; 95% CI = 0.88–1.06). Similarly, Wallin et al. (2022) did not find statistical evidence that universal background checks, point-of-contact background check laws, or permit-to-purchase requirements affected rates of IPH or firearm-related IPH among black or white victims. Estimates from both studies have serious methodological concerns as these policies were not the focus of the research but were instead included as controls for potential confounds, and thus their effect sizes are unlikely to have a valid causal interpretation (Hünermund and Louw 2022).

Finally, two studies with serious methodological concerns evaluated waiting period requirements. Edwards et al. (2018) found that waiting period requirements significantly reduced firearm-related suicides overall (IRR = 0.98, 95% CI = 0.97–1.00). Stratifying by white versus nonwhite victims, relative effect sizes were similar (white IRR = 0.98, 95% CI = 0.96–1.00; nonwhite IRR = 0.94; 95% CI = 0.87–1.02) and did not significantly differ from each other in rate ratio (*p* = 0.38) or rate difference terms (*p* = 0.42). Hepburn et al. (2004) did not find significant effects of handgun waiting period requirements on homicide rates overall (IRR = 0.94; 95% CI = 0.86–1.27) or among white males ages 35 and older (IRR = 0.99; 95% CI = 0.89–1.11).

### Policies regulating the legal use, storage, and carrying of firearms

Four studies (D’Alessio et al. 2022; Degli Esposti et al. 2022; Knopov et al. 2019; McClellan and Tekin 2017) evaluated whether stand-your-ground (SYG) laws produced differential effects by racial groups. Two of these studies had at least one outcome rated as high methodological quality (Degli Esposti et al. 2022; McClellan and Tekin 2017), one had minor concerns related to identifying assumptions and outcome missingness (Knopov et al. 2019), and one had serious concerns related to causal identification and statistical assumptions (D’Alessio et al. 2022).

Based on monthly mortality data from 1999 to 2017, Degli Esposti et al. (2022) estimated the relationship of SYG laws with homicide and firearm homicide rates. Their results showed that SYG laws significantly increased rates of homicide and firearm-specific homicide by about 8%, with similar findings across a range of sensitivity analyses, including alternative modeling frameworks. Estimates stratified by race showed no significant differences from each other (either in rate ratio [*p* = 0.19] or rate difference [*p* = 0.34] terms), with effects on total homicide rates indicating 10% increases for white victims (IRR = 1.10; 95% CI = 1.05–1.15) and 5% increases for nonwhite victims (IRR = 1.05, 95% CI = 1.00–1.11); effects specific to firearm homicides were similar. Results from the analysis of annual mortality data from 1991 to 2016 in Knopov et al. (2019), which dropped eleven states with low counts of black homicide victims, also showed no significant differences by race in the effects of SYG laws, with both groups experiencing small and statistically insignificant increases in homicide rates (IRR = 1.03; 95% CI = 0.97–1.09).

Covering a shorter timeframe, 2000 to 2010, McClellan and Tekin (2017) found significant increases in monthly firearm homicide rates with SYG law adoption (IRR = 1.08; 95% CI = 1.01–1.15). Their race-specific results differ from Degli Esposti et al. (2022) in that effects were significant for white homicide victims (IRR = 1.22; 95% CI = 1.11–1.34) but not nonwhite homicide victims (IRR = 0.98; 95% CI = 0.83–1.15). Based on their Poisson specification, increases in firearm homicides among white victims were significantly larger than effects among nonwhite victims in rate ratio terms (*p* = 0.02) but not in rate difference terms (*p* = 0.55). Larger and more precisely estimated relative effects for whites were also found when distinguishing non-justifiable and justifiable homicides, as well as for analyses focused on firearm assault injury hospitalizations and emergency department visits. However, these additional analyses have critical concerns related to model overfit.

Based on data from 2002 to 2015 covering 95 cities across 15 states, D’Alessio et al. (2022) found that SYG laws significantly increased the percentage of reported crimes involving firearms, with interaction terms showing significantly larger increases in states where a higher proportion of firearm crimes involve black offenders (*p* = 0.004). However, this study’s regression model controls for a variety of measures related to the composition of firearm crimes that are conceivably affected by the passage of SYG laws (e.g., the percentage of firearm crimes involving young offenders, involving black offenders, or occurring in residences), creating serious concerns about the validity and interpretation of the study’s results.

Five studies evaluated effects of concealed carry laws. Two studies with minor methodological concerns estimated effects of shall-issue laws on homicides (Hepburn et al. 2004; Knopov et al. 2019). Using data from 1979 to 1998, Hepburn et al. (2004) found no significant effect of shall-issue laws on homicide rates overall (IRR = 1.01; 95% CI = 0.94–1.10) or among white males ages 35 and older (IRR = 1.03; 95% CI = 0.94–1.11). Based on more recent data spanning 1991 to 2016, Knopov et al. (2019) instead found that shall-issue laws significantly increased homicide rates overall (IRR = 1.06, 95% CI = 1.04–1.11); while they found no significant effect differences for white or black individuals in rate ratio terms, larger increases in homicides of black individuals are significant in rate difference terms (*p* = 0.01). While they separately estimated effects of permitless carry laws, these results were highly imprecise and based on law changes in a small number of states, which raises serious methodological concerns.

Two other studies (Lott and Mustard 1997; Olson and Maltz 2001) evaluating effects of shall-issue laws on the racial composition of violent crime types showed highly imprecise effects and were subject to serious methodological concerns due to failure to account for serial correlation. Another study (Rubin and Dezhbakhsh 2003) evaluated whether counties’ racial composition moderated the effects of shall-issue laws on violent crime by treating predicted changes in violent crime due to shall-issue law adoption (as estimated through prior regression models) as the dependent variable in a regression with a variety of independent variables measuring county characteristics, such as population density and state racial composition. While results showed significant relationships between race variables and predicted effects on violent crime, the direction of these effects varied (e.g., number of black males aged 10–29 had a significant negative estimate, while number of black females aged 10–29 had a significant positive estimate), and there are critical methodological concerns such that these estimates should not be taken as causal as well as serious statistical violations that likely resulted in underestimated standard errors.

Finally, one study (Anderson et al. 2021) with minor methodological concerns found that child access prevention laws significantly reduced firearm murders involving white juvenile offenders (IRR = 0.77, 95% CI = 0.62–0.95). Effects for black juvenile offenders were negative but not significantly different from zero (IRR = 0.89, 95% CI = 0.75–1.05) nor significantly different from effects for white offenders in either rate ratio (*p* = 0.30) or rate difference (*p* = 0.35) terms.

### Summary of studies without serious methodological concerns

**Fig. 2:**
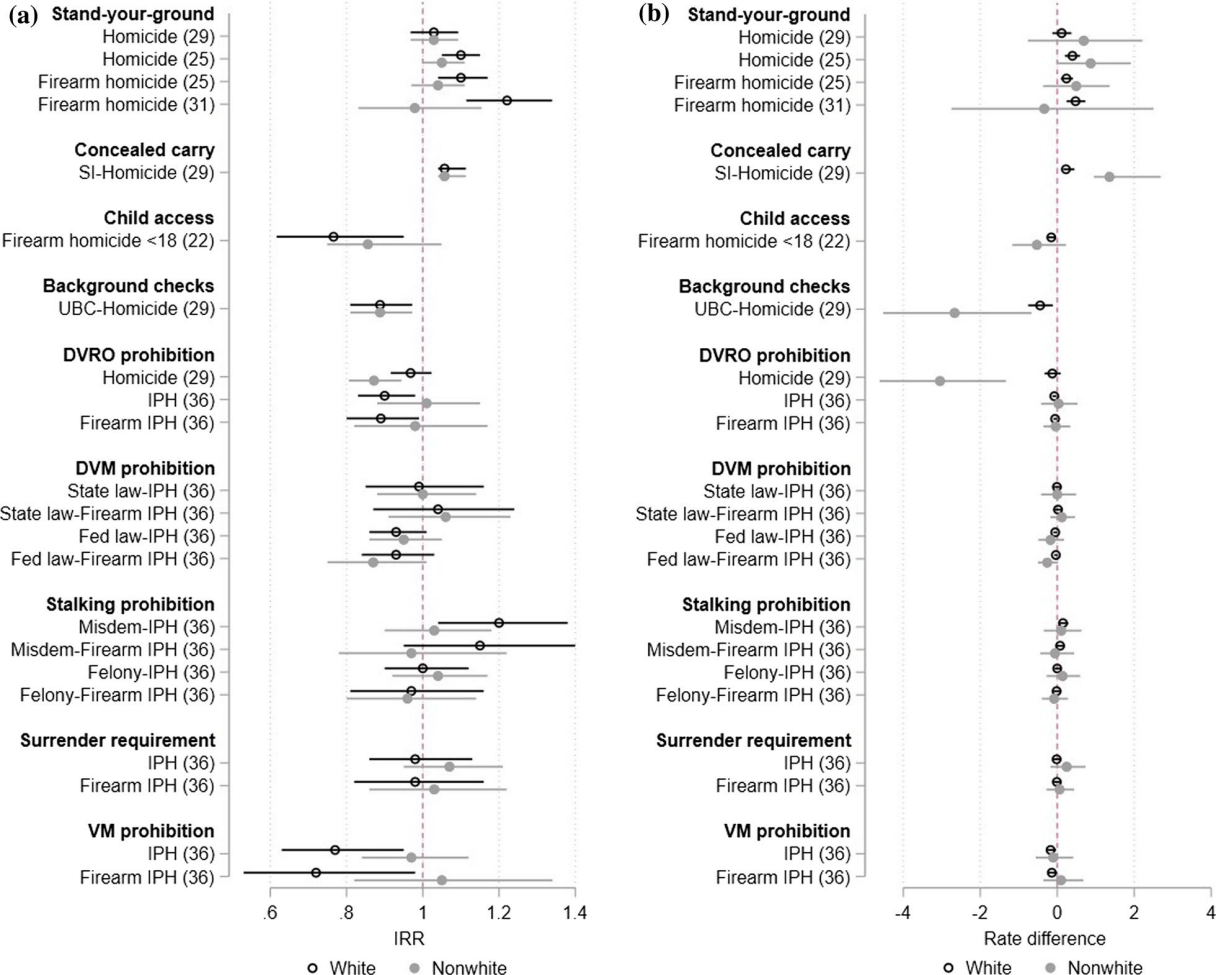
Policy effects by race and ethnicity among studies without serious or critical methodological concerns. **a** Policy effects in rate ratio terms. **b** Policy effects in rate difference terms. Notes: IRR, incidence rate ratio. DVRO, domestic violence restraining order. IPH, intimate partner homicide. DVM, domestic violence misdemeanor. Misdem, Misdemeanor. SI, shall-issue. VM, violent misdemeanor. UBC, universal background check. Racial groups were defined differently across studies (see details in Table 1) and the distinction between white and nonwhite used in the legend is thus a crude characterization. Parenthetical numbers represent the study citation

Figure 2 presents effect comparisons from analyses that we did not consider as having serious or critical methodological concerns. Figure 2a presents effects in IRR terms, which are those estimates provided in the studies themselves (or, in the case of log-linear models, approximated by exponentiating the coefficient estimate). Figure 2b instead uses estimates of mean incidence rates by race/ethnicity to convert relative effect estimates provided by each study into effect estimates in terms of incidence rate differences (see Additional file 1: Appendix D and Table D.2 for details on sources of mean incidence rates and calculations for estimate conversions).

## Conclusions

Precisely because blacks are at such higher risk of firearm violence, in part due to the cumulative effects of structural racism, they have much more to potentially gain from any law that reduces firearm violence. On the other hand, it is possible that—also due to structural racism—the laws are structured or enforced in a way that prioritizes minimizing harms for white residents of the state. In that case you might expect black people and communities to benefit less from laws that reduce violence. It is not clear how these two competing factors play out in the real world, but both could be explained as a product of structural racism.

The existing literature has largely not found evidence that firearm policies have significantly different relative effects on firearm injury or violent crime across racial or ethnic groups. This does not imply that such differential effects are rare or even small. Instead, efforts to study such effects have yet to overcome important barriers. For decades, the USA has underinvested in firearm violence and firearm policy research, and thus the relative dearth of studies evaluating differential racial and ethnic impacts of firearm policies may in part reflect broader barriers faced by the field. Indeed, of the 15 studies we identified, fewer than half formally tested such differences. In addition, the relatively narrow set of outcomes evaluated (e.g., death and crime) as well as the broad race/ethnicity category designations (e.g., white vs nonwhite) employed by researchers highlight another barrier to understanding the equity implications of US firearm policy, but these limitations are understandable. For cross-jurisdiction comparisons, mortality datasets represent one of the only sources of outcome data that can provide comparable and relatively complete information on race/ethnicity, and deaths are sufficiently rare that large race groupings may be necessary to detect differential effects. Evaluating higher-prevalence outcomes such as hospitalizations can improve power, but it often comes at the cost of having to restrict analyses to a small number of states or a single state (McClellan and Tekin 2017; Pear et al. 2022). Building data infrastructure and improving statistical power are serious challenges that are unlikely to be addressed in the short-term (Roman and Cook 2021; Morral and Smart 2022; Barber et al. 2022).

While subject to the aforementioned considerations, of the 57 effect comparisons that met our inclusion criteria, a few did suggest that the relative effects of firearm policies vary across racial or ethnic groups. While one study found that stand-your-ground laws produced significantly greater increases in firearm homicide rates among white victims compared to black victims, this did not replicate in a subsequent study using similar methods but assessing a longer timeframe with eight more years of data.

Studies also showed some evidence of significantly different relative effects of policies prohibiting firearm possession among individuals with DVROs or violent misdemeanors. However, the results of these studies are puzzling, even when excluding studies with more serious risk of bias. One study indicated that some laws prohibiting firearm purchase and possession based on civil or criminal documentation of violence risk are significantly more effective in producing relative reductions in IPH among white compared to nonwhite individuals. However, another study that evaluated effects on homicide more broadly found that these types of policies produced significantly greater relative (and absolute) benefits for black victims compared to white victims. Several factors might explain these seemingly contradictory findings, including differences in how policies influence intimate partner versus stranger homicides. Furthermore, because the studies had different geographic coverage and evaluated different timeframes, different findings may reflect differences in implementation of these laws that are associated with local demographic characteristics. It is well established, for instance, that even when required by law to order the removal of firearms from subjects of DVROs, courts are inconsistent in their compliance with this rule (Zeoli et al. 2022).

Even when there are no differences in the relative effects of laws, the absolute effect can differ significantly by race, due to large racial disparities in homicide and firearm homicide rates that are the consequence of structural racism and economic segregation (Multiple Cause of Death by Single Race 2018–2021 on CDC WONDER Online Database 2022). In these cases, there may be no evidence that a law is inconsistently applied across racial groups, but one or more groups disproportionately benefit or suffer from the law’s effects. Indeed, when we convert risk ratio estimates to per capita rate differences, we find evidence that some policies regulating firearm sales and transfers (universal background check requirements and permit-to-purchase licensing regimes for handguns) produce significantly greater reductions in homicide rates among black compared to white populations. These policies do not have as clear a racial valence as some other firearm regulations—such as prohibiting firearm possession based on criminal history, an outcome inextricably linked to racially unjust criminal justice systems—but they may differentially affect black populations through increasing costs of transacting in the types of informal or illegal firearm markets that contribute to the gun violence disproportionately experienced by communities of color (Braga and Cook 2023). In contrast, permissive shall-issue concealed carry laws are associated with significantly greater increases in firearm homicide rates among black compared to white victims. Here too, by reducing the burden of obtaining a concealed carry permit and by removing law enforcement discretion from the permit issuance decision, these laws may affect the number and composition of firearm owners and carriers in ways that differentially affect firearm homicide victimization risk for black individuals.

Based on the overall methodological quality of the literature we reviewed, there are several opportunities for improvement. First, as noted, few studies explicitly test for significance of differences by race or ethnicity; instead, it is common to conduct stratified analyses and note that estimates for one group were significant and for the other were not. This does not, of course, imply that the two estimates are meaningfully different from each other. Future studies could be improved with clearer conceptual motivation for whether policies are expected to differentially affect different racial/ethnic group in relative or absolute terms and consideration of whether one or both of these differences is of policy relevance. Finally, studies need to be more transparent about the quality and completeness of the race/ethnicity data used. Degli Esposti et al. (2022) offer an excellent example of this type of transparent reporting, which can help improve subsequent research using these same data. Research that centers evaluation of racial and ethnic differences in the effects of firearm policy as its primary focus (Ford and Airhihenbuwa 2010), rather than as a series of subgroup analyses or secondary heterogeneity analyses, is needed to rigorously inform the distributional consequences of changes in firearm policy.

Even with such efforts, identification of interactions between law effects and population subgroups is likely to be challenging because the main effects of laws are likely to be small (perhaps changing outcomes by 5% or less) and because many laws are implemented at the state level, meaning there are only 50 analytic units in every year. These factors combined mean analyses of disparate law effects may inevitably suffer from low statistical power. One approach to addressing this problem and for reducing the incidence of statistically significant effects with exaggerated magnitudes or that provide misleading evidence about even the direction of the effect would be to analyze disparities in law effects using a Bayesian approach. Integrating qualitative assessments with quantitative analyses may support robustness of findings as well as better contextualize the ways in which firearm policies interact with broader structural and systemic factors that contribute to levels of and disparities in firearm violence (Buggs et al. 2023).

### Limitations

Our review of research on the differential effects of laws by race examines only a limited set of outcomes potentially associated with firearm laws. It did not examine research on their implementation or enforcement, which could represent an important source of disparate harms of more restrictive firearm regulations given well-documented racial and ethnic disparities present at multiple points in the criminal legal system (National Academies of Sciences Engineering and Medicine 2022). Some such harms, like incarceration, would not be well captured by the outcomes we investigated. Additionally, for the outcomes we considered, most studies did not test for significance of differences by race and thus we had to calculate this information under assumptions of independence.

### Public health implications

This study demonstrates that, despite growing evidence that some restrictive firearm laws can reduce and some permissive firearm laws can increase firearm-related violence in aggregate, we know little about firearm laws’ differential effects across racial groups in the USA. Although there is some evidence that a few laws may have different relative or absolute effects by race groups, few studies have provided evidence on this question, and fewer still have explicitly focused on it. Because of the enormous racial and ethnic disparities in the harms of firearm violence, it is important to identify whether policies reduce or exacerbate such disparities. More rigorous and more targeted study of the differential effects of firearm laws by race are needed.

### Supplementary Information

**Additional file 1. **Details on study methods and results.

## Data Availability

The datasets used and/or analyzed during the current study are available from the corresponding author on reasonable request.

## Abbreviations

CI: Confidence interval
DV: Domestic violence
DVRO: Domestic violence restraining order
ERPO: Extreme risk protection order
IPH: Intimate partner homicide
IRR: Incidence rate ratio
ROBINS-I: Risk Of Bias In Non-randomized Studies-of Interventions
