# Supplementary material for: Racial and ethnic differences in the effects of state firearm laws: a systematic review subgroup analysis
Source: Inj Epidemiol. 2023 Dec 14;10:67. doi: 10.1186/s40621-023-00477-y (PMC10722776; doi:10.1186/s40621-023-00477-y)
Supplement: Supplementary file 1 — Additional file 1. Details on study methods and results. [file 40621_2023_477_MOESM1_ESM.docx]

**ONLINE-ONLY SUPPLEMENTAL MATERIAL**

Appendix A: Details on Search Strategy, Inclusion and Exclusion Criteria, and Data Extraction

Appendix B: Full Search String for Scopus

Appendix C: Details on Quality Assessment Domains

Appendix D: Details on Included Studies and Quality Assessment Results

**Appendix A: Details on Search Strategy, Inclusion and Exclusion Criteria, and Data Extraction**

**Literature review and included study details**

This review and meta-analysis drew from a broader systematic review of the effects of 18 classes of gun policies on eight outcomes (Smart et al., in progress), which updated and expanded our earlier systematic review (Smart et al., 2023). The policies and outcomes included in the broader review were:

- 1. Policies
     1. Policies regulating who may legally own, purchase, or possess firearms
        1. minimum age requirements
        2. prohibitions associated with mental illness
        3. prohibitions associated with domestic violence
        4. surrender of firearms by prohibited possessors
        5. extreme risk protection orders
     2. Policies regulating the sale and transfer of firearms
        1. background checks
        2. licensing and permitting requirements
        3. waiting periods
        4. firearm safety training requirements
        5. lost or stolen firearm reporting requirements
        6. firearm sales reporting, recording, and registration requirements
        7. bans on the sale of assault weapons and high-capacity magazines
        8. bans on low-quality handguns (i.e., “junk gun” or “Saturday night special” bans)
     3. Policies regulating the legal use, storage and carrying of firearms
        1. stand-your-ground laws
        2. child-access prevention laws
        3. concealed-carry laws
        4. gun-free zones
        5. laws allowing armed staff in kindergarten through grade 12 (K-12) schools
  2. Outcomes
     1. suicide
     2. violent crime (e.g., homicides, assaults, robberies)
     3. unintentional injuries and deaths
     4. mass shootings
     5. officer-involved shootings
     6. defensive gun use
     7. hunting and recreation
     8. gun industry outcomes (e.g., revenues, employment)

For the broader systematic review, we considered all policy-outcome combinations; for the current study, we similarly considered all policy-outcome combinations. While certain policies and outcomes may have more or less relevance to racial and ethnic disparities, we did not adopt theoretical criteria for evaluating which policies and outcomes to include.

**Searches**

We searched the following databases: PubMed, APA PsycInfo, Index to Legal Periodicals and Books, Social Science Abstracts, Web of Science, Criminal Justice Abstracts, National Criminal Justice Reference Service, Sociological Abstracts, EconLit, Business Source Complete, WorldCat, Scopus, LawReviews (LexisNexis).

For feasibility, we restricted our search to English-language articles. In all, the search timeframe covered January 1, 1995 through February 28, 2023. During our review, we became aware that a newer version of a working paper captured in our search (Luca et al., 2016) had been published in a peer-review journal (Luca et al., 2020) and inadvertently treated as a duplicate reference. We now treat Luca et al. (2020) as superseding Luca et al. (2016), although neither meets inclusion criteria for this subgroup analysis from the broader systematic review.

We conducted separate searches for each of the eight outcomes. The search strings that were applied universally across all outcomes are:

- gun OR guns OR firearm* OR handgun* OR shotgun* OR rifle* OR longgun* OR machinegun* OR pistol* OR “automatic weapon*” OR “assault weapon*” OR “semi-automatic weapon*” OR “semiautomatic weapon*” OR “Saturday night special*”

AND

- ownership OR own OR owns OR availab* OR access* OR possess* OR purchas* OR restrict* OR regulat* OR distribut* OR “weapon carrying” OR “weapon-carrying” OR legislation OR legislating OR legislative OR law OR laws OR legal* OR policy OR policies OR ban OR bans OR banned OR “concealed carry”

AND each of the following outcome-specific search terms:

1. suicide: suicid* OR “self-harm*” OR “self-injur*” OR “self injur*”;
2. violent crime: homicide* OR murder* OR manslaughter OR “domestic violence” OR “spousal abuse” OR “spouse abuse” OR “elder abuse” OR “child abuse” OR “family violence” OR “child maltreatment” OR “spousal maltreatment” OR “elder maltreatment” OR “elder mistreatment” OR “intimate relationship violence” OR “intimate partner violence” OR “dating violence” OR (violen* AND [crime* OR criminal*]) OR rape OR rapes OR rapist* OR “personal crime” OR “personal crimes” OR robbery OR robberies OR assault* OR stalk* OR terroris*
3. unintentional injuries and deaths: accident* OR unintentional* OR “un intentional*”
4. mass shootings: “mass shooting*” OR “school shooting*”
5. officer-involved shootings: “law enforcement” OR police* OR policing (for some databases also include: OR “use of force” OR “deadly force”)
6. defensive gun use: self-defense OR “self defense” OR “personal defense” OR defens* OR “self-protect*” OR “self protect*” OR DGU OR SDGU
7. hunting and recreation: hunt OR hunting OR “sport shooting” OR “shooting sports” OR recreation*
8. gun industry: industr* OR manufactur* OR produc* OR distribut* OR supply OR supplies OR trade OR trading OR price* OR export* OR revenue* OR sales OR employ* OR profit* OR cost OR costs OR costing OR “gun show” OR tax OR taxes OR taxing OR taxation OR payroll OR “federal firearms license” OR FFL

The full electronic search strategy for Scopus is presented in Appendix B.

**Inclusion and Exclusion Criteria**

Our inclusion criteria restrict to studies that estimated an effect of one of our 18 classes of gun policy and used research methods designed to identify causal effects among observed associations between policies and outcomes. Specifically, we required, at a minimum, that studies include time-series data and use such data to establish that policies preceded their apparent effects and that studies include a control group or comparison group in model estimation (to demonstrate that the purported causal effect was not found among those who were not exposed to the policy). On a case-by-case basis, we examined studies that made a credible claim to causal inference on the basis of data that did not include a time series or that did not include a control group of individuals or jurisdictions unexposed to the policy. We excluded studies if they were commentaries or conceptual discussions, systematic reviews or meta-analyses, case studies, dissertations, conference abstracts, legal statutes or congressional hearings, or purely descriptive studies. These inclusion criteria were designed to restrict to studies that plausibly estimated a causal estimate of a policy effect on firearm homicides or suicides, and that also estimated how that policy shock influenced total or non-firearm homicides or suicides.

For the purposes of this study, we further required that the article investigated and provided information on policy effects differentiated for subpopulations defined by race or ethnicity.

One study (Humphreys et al., 2017) modeled the effects of Florida’s stand-ground-law in Florida in models stratified for White versus African American individuals; however, unlike the primary aggregate analysis, the stratified analyses did not include a comparison group of states and thus did not meet our inclusion criteria. We also exclude the firearm homicide estimates from Knopov et al. (2019) as insufficient information on inferential statistics was provided.

**Data Extraction**

Table A.1 presents the data items extracted from each study.

**Table A.1. List of Item Fields in Data Extraction Form**

| **Category** | **Data item** |
| --- | --- |
| General source information | Author (year) |
|  | Title |
|  | Article funding source |
| Methods | Geographic unit of analysis |
|  | Temporal unit of analysis |
|  | Model specification (e.g., link function) |
|  | Use of population weights |
|  | Control for national time trends |
|  | Control for unit-specific effects (e.g., state fixed effects) |
|  | Control for unit-specific trends |
|  | Inclusion of autoregressive effects |
|  | Other covariates included |
|  | Standard error calculation or adjustment |
|  | Other notes |
| Outcome information | Outcome category |
|  | Specific outcome |
|  | Population restrictions (e.g., age, gender) |
|  | Time period studied |
|  | Outcome data source |
| Policy information | Policy category |
|  | “Treated” condition |
|  | “Control” condition |
|  | Number of treated units |
| Effect size estimates | Source of estimate |
|  | Source explanation (i.e., why this estimate was chosen) |
|  | Form of estimate provided |
|  | Linear model coefficient estimate (if provided) |
|  | Linear model coefficient standard error (if provided) |
|  | IRR (if provided) |
|  | Test statistic (if provided) |
|  | p-value (if provided) |
|  | Coefficient lower 95% confidence interval (if provided) |
|  | Coefficient upper 95% confidence interval (if provided) |
|  | IRR lower 95% confidence interval (if provided) |
|  | IRR upper 95% confidence interval (if provided) |
|  | Authors test for difference by race/ethnicity |
| Additional information for quality assessment | Number of observations |
|  | Number of events |
|  | Number of parameters |
|  | Notes on assessing potential for model overfit |
|  | Notes on assessing statistical assumptions |
|  | Notes on assessing causal identification assumptions |
|  | Other concerns (e.g., model sensitivity) |

**Search Results**

Of the 182 articles that underwent full text review, 15 met our inclusion criteria and estimated the effect of one our policies of interest on an included outcome among subpopulations defined by race or ethnicity (see Table A.2 for the full list of studies that underwent full text review for this subgroup analysis and were subsequently screened out as excluded).

**Table A.2. Studies that underwent full text review and reasons for exclusion**

| **No.** | **Study** | **Included** | **Reason for Exclusion** |
| --- | --- | --- | --- |
| 1 | Alexander AK, “No Retreat: The Impact of Stand Your Ground Laws on Violent Crime,” *Criminal Justice Review*, 2022. | NO | No analysis by race/ethnicity |
| 2 | Anderson DM, Sabia JJ, “Child-Access-Prevention Laws, Youths’ Gun Carrying, and School Shootings,” J*ournal of Law and Economics*, Vol. 61, No. 3, August 2018, pp. 489–524. | NO | No analysis by race/ethnicity |
| 3 | Anderson, DM, Sabia JJ, Tekin E. *Child Access Prevention Laws and Juvenile Firearm-Related Homicides.* Cambridge, Mass.: National Bureau of Economic Research, Working Paper No. 25209, December 2018. | NO | Superseded by Anderson et al. (2021) |
| 4 | Anderson, DM, Sabia JJ, Tekin E. “Child Access Prevention Laws and Juvenile Firearm-Related Homicides,” *Journal of Urban Economics*, Vol. 126, 2021, 103387. | YES |  |
| 5 | Andrés AR, and Hempstead K. “Gun Control and Suicide: The Impact of State Firearm Regulations in the United States, 1995–2004,” Health Policy, Vol. 101, No. 1, 2011, pp. 95–103. | NO | No analysis by race/ethnicity |
| 6 | Aneja A, Donohue III JJ, Zhang A. *The Impact of Right to Carry Laws and the NRC Report: The Latest Lessons for the Empirical Evaluation of Law and Policy*. NBER Working paper; 2012. | NO | No analysis by race/ethnicity |
| 7 | Aneja A, Donohue III JJ, Zhang A. *The Impact of Right to Carry Laws and the NRC Report: The Latest Lessons for the Empirical Evaluation of Law and Policy*. Stanford, Calif.: Stanford Law School, Olin Working Paper No. 461; 2014. | NO | No analysis by race/ethnicity |
| 8 | Anestis MD, Khazem LR, Law KC, Houtsma C, LeTard R, Moberg F, Martin R. The association between state laws regulating handgun ownership and statewide suicide rates. *Am J Public Health*. 2015;105(10): 2059-67. | NO | No analysis by race/ethnicity |
| 9 | Ayres I, Donohue JJ. Nondiscretionary concealed weapons laws: a case study of statistics, standards of proof, and public policy. *Am Law Econ Rev*. 1999;1(1): 436-470. | NO | No analysis by race/ethnicity |
| 10 | Ayres I, Donohue JJ. Shooting down the "more guns, less crime" hypothesis. *Stanford Law Rev*. 2003;55(4): 1193-1312. | NO | No analysis by race/ethnicity |
| 11 | Ayres I, Donohue JJ. *Shooting Down the More Guns, Less Crime Hypothesis*. NBER Working Paper; 2002. | NO | No analysis by race/ethnicity |
| 12 | Ayres I, Donohue JJ. The latest misfires in support of the more guns, less crime hypothesis. *Stanford Law Rev*. 2003;55: 1371-1398. | NO | No analysis by race/ethnicity |
| 13 | Ayres I, Donohue JJ. Yet another refutation of the more guns, less crime hypothesis - With some help from Moody and Marvell. Econ Journal Watch. 2009;6(1): 35-59. | NO | No analysis by race/ethnicity |
| 14 | Ayres I, Donohue, JJ. More guns, less crime fails again: The latest evidence from 1977-2006. *Econ Journal Watch*. 2009;6(2): 218-U63. | NO | No analysis by race/ethnicity |
| 15 | Azad HA, Monuteaux MC, Rees CA, Siegel M, Mannix R, Lee LK, Sheehan KM, Fleegler EW. “Child Access Prevention Firearm Laws and Firearm Fatalities Among Children Aged 0 to 14 Years, 1991–2016,” JAMA Pediatrics, Vol. 174, No. 5, May 2020, pp. 463–469. | NO | No analysis by race/ethnicity |
| 16 | Balakrishna M, Wilbur KC. “Do Firearm Markets Comply with Firearm Restrictions? How the Massachusetts Assault Weapons Ban Enforcement Notice Changed Registered Firearm Sales,” Journal of Empirical Legal Studies, Vol. 19, No. 1, 2022, pp. 60–89. | NO | No analysis by race/ethnicity |
| 17 | Barati M. New evidence on the impact of concealed carry weapon laws on crime. *International Review of Law and Economics*. 2016;47: 76-83. | NO | No analysis by race/ethnicity |
| 18 | Bartley WA, Cohen MA. The effect of concealed weapons laws: an extreme bound analysis. *Economic Inquiry*. 1998;36(2): 258-265 | NO | No analysis by race/ethnicity |
| 19 | Benson BL, Mast BD. Privately produced general deterrence. *The Journal of Law and Economics*. 2001;44(S2): 725-746. | NO | No analysis by race/ethnicity |
| 20 | Bhatt, Apurva, Xi Wang, An-Lin Cheng, Kalee L. Morris, Luke Beyer, Abbie Chestnut, Kristy Steigerwalt, and Jeffrey Metzner, “Association of Changes in Missouri Firearm Laws with Adolescent and Young Adult Suicides by Firearms,” JAMA Network Open, Vol. 3, No. 11, 2020, pp. e2024303–e2024303. | NO | No analysis by race/ethnicity |
| 21 | Black DA, Nagin D. Do right-to-carry laws deter violent crime? *Journal of Legal Studies*. 1998;27: 209-291. | NO | No analysis by race/ethnicity |
| 22 | Blau BM, Gorry DH, Wade C. Guns, laws and public shootings in the United States. *Applied Economics*. 2016;48(49): 4732-4746. | NO | No analysis by race/ethnicity |
| 23 | Brauer J, Montolio D, Trujillo-Baute E. How do US state firearms laws affect firearms manufacturing location? An empirical investigation, 1986-2010. *Journal of Economic Geography*. 2017;17(4): 753-790. | NO | No analysis by race/ethnicity |
| 24 | Bronars SG, Lott Jr JR. Criminal deterrence, geographic spillovers, and the right to carry concealed handguns. *American Economic Review*. 1998;88(2): 475-479 | NO | No analysis by race/ethnicity |
| 25 | Castillo-Carniglia, Alvaro, Rose M. C. Kagawa, Magdalena Cerdá, Cassandra K. Crifasi, Jon S. Vernick, Daniel W. Webster, and Garen J. Wintemute, “California’s Comprehensive Background Check and Misdemeanor Violence Prohibition Policies and Firearm Mortality,” Annals of Epidemiology, Vol. 30, February 2019, pp. 50–56. | NO | No analysis by race/ethnicity |
| 26 | Cheng C, Hoekstra M. Does strengthening self-defense law deter crime or escalate violence? Evidence from expansions to Castle Doctrine. *Journal of Human Resources*. 2013;48(3): 821-854. | NO | No analysis by race/ethnicity |
| 27 | Chien, L-C., and M. Gakh, “The Lagged Effect of State Gun Laws on the Reduction of State-Level Firearm Homicide Mortality in the United States from 1999 to 2017,” Public Health, Vol. 189, 2020, pp. 73–80. | NO | No analysis by race/ethnicity |
| 28 | Cook PJ, Ludwig J. The effects of the Brady Act on gun violence. In: Guns, Crime, and Punishment in America, B Harcourt, ed. New York University Press; 2003: 283-98. | NO | No analysis by race/ethnicity |
| 29 | Crifasi CK, Meyers JS, Vernick JS, Webster DW. Effects of changes in permit-to-purchase handgun laws in Connecticut and Missouri on suicide rates. *Prev Med*. 2015;79: 43-9. | NO | No analysis by race/ethnicity |
| 30 | Crifasi CK, Merrill-Francis M, McCourt A, Vernick JS, Wintemute GJ, Webster DW. Association between firearm laws and homicide in urban counties. *J Urban Health.* 2018;95(3):383-390. | NO | No analysis by race/ethnicity |
| 31 | Crifasi, Cassandra K., Keshia M. Pollack, and Daniel W. Webster, “Effects of State-Level Policy Changes on Homicide and Nonfatal Shootings of Law Enforcement Officers,” Injury Prevention, Vol. 22, No. 4, 2016, pp. 274–278. | NO | No analysis by race/ethnicity |
| 32 | Cummings P, Grossman DC, Rivara FP, Koepsell TD. State gun safe storage laws and child mortality due to firearms. *J Am Med Assoc.* 1997;278(13):1084-1086. | NO | No analysis by race/ethnicity |
| 33 | D’Alessio, Stewart J., Lisa Stolzenberg, Rob T. Guerette, and Kristen M. Zgoba, “The Effect of Self-Defense Laws on Firearm Use Among Criminal Offenders,” Crime & Delinquency, 2022. | YES |  |
| 34 | Dalafave, Rachel, “An Empirical Assessment of Homicide and Suicide Outcomes with Red Flag Laws,” Loyola University Chicago Law Journal, Vol. 52, 2020, pp. 867–904. | YES |  |
| 35 | Degli Esposti, Michelle, Douglas J. Wiebe, Antonio Gasparrini, and David K. Humphreys, “Analysis of ‘Stand Your Ground’ Self-Defense Laws and Statewide Rates of Homicides and Firearm Homicides,” JAMA Network Open, Vol. 5, No. 2, 2022, pp. e220077–e220077. | YES |  |
| 36 | Depew, Briggs, and Isaac Swensen, “The Effect of Concealed-Carry and Handgun Restrictions on Gun-Related Deaths: Evidence from the Sullivan Act of 1911,” The Economic Journal, Vol. 132, No. 646, 2022, pp. 2118–2140. | NO | No analysis by race/ethnicity |
| 37 | DeSimone J, Markowitz S. *Effect of child access prevention laws on non-fatal gun injuries*. NBER Working Paper; 2005. | NO | No analysis by race/ethnicity |
| 38 | DeSimone, J., S. Markowitz, and J. Xu, “Child Access Prevention Laws and Nonfatal Gun Injuries,” Southern Economic Journal, Vol. 80, No. 1, 2013, pp. 5–25. | NO | No analysis by race/ethnicity |
| 39 | Dezhbakhsh H, Rubin PH. Lives saved or lives lost? The effects of concealed-handgun laws on crime. *American Economic Review*. 1998;88(2): 468-474 | NO | No analysis by race/ethnicity |
| 40 | Díez C, Kurland RP, Rothman EF, Bair-Merritt M, Fleegler E, Xuan ZM, Galea S, Ross CS, Kalesan B, Goss KA, Siegel M. State intimate partner violence-related firearm laws and intimate partner homicide rates in the United States, 1991 to 2015. *Annals of Internal Medicine*. 2017;167(8). | NO | No analysis by race/ethnicity |
| 41 | Donohue JJ. Impact of Concealed-Carry Laws [From Evaluating Gun Policy: Effects on Crime and Violence, P 287-341, 2003, Philip J. Cook and Jens Ludwig, eds.]; 2003. | NO | No analysis by race/ethnicity |
| 42 | Donohue JJ. Guns, crime, and the impact of state right-to-carry laws. *Fordham Law Review*; 2004. | NO | No analysis by race/ethnicity |
| 43 | Donohue JJ. Laws Facilitating Gun Carrying and Homicide. *Am J Public Health.* 2017;107(12):1864-1865. | NO | No analysis by race/ethnicity |
| 44 | Donohue, John J., Abhay Aneja, and Kyle D. Weber, Right-to-Carry Laws and Violent Crime: A Comprehensive Assessment Using Panel Data and a State-Level Synthetic Control Analysis, Cambridge, Mass.: National Bureau of Economic Research, Working Paper No. 23510, November 2018. | NO | No analysis by race/ethnicity |
| 45 | Donohue JJ, Aneja A, Weber KD. Right‐to‐Carry Laws and Violent Crime: A Comprehensive Assessment Using Panel Data and a State‐Level Synthetic Control Analysis. *Journal of Empirical Legal Studies.* 2019;16(2):198-247. | NO | No analysis by race/ethnicity |
| 46 | Donohue, John J., and Steven D. Levitt, “The Impact of Legalized Abortion on Crime,” Quarterly Journal of Economics, Vol. 116, No. 2, 2001, pp. 379–420. | NO | No analysis by race/ethnicity |
| 48 | Donohue, John J., Samuel V. Cai, Matthew V. Bondy, and Philip J. Cook, More Guns, More Unintended Consequences: The Effects of Right-to-Carry on Criminal Behavior and Policing in US Cities, Cambridge, Mass.: National Bureau of Economic Research, Working Paper No. 30190, June 2022. | NO | No analysis by race/ethnicity |
| 47 | Doucette, Mitchell L., Cassandra K. Crifasi, and Shannon Frattaroli, “Right-to-Carry Laws and Firearm Workplace Homicides: A Longitudinal Analysis (1992–2017),” American Journal of Public Health, Vol. 109, No. 12, 2019, pp. 1747–1753. | NO | No analysis by race/ethnicity |
| 49 | Doucette, Mitchell L., Christa Green, Jennifer Necci Dineen, David Shapiro, and Kerri M. Raissian, “Impact of ShotSpotter technology on Firearm Homicides and Arrests among Large Metropolitan Counties: A Longitudinal Analysis, 1999–2016,” Journal of Urban Health, Vol. 98, No. 5, 2021, pp. 609–621. | NO | No analysis by race/ethnicity |
| 50 | Doucette, Mitchell L., Julie A. Ward, Alex D. McCourt, Daniel Webster, and Cassandra K. Crifasi, “Officer-Involved Shootings and Concealed Carry Weapons Permitting Laws: Analysis of Gun Violence Archive Data, 2014–2020,” Journal of Urban Health, Vol. 99, No. 3, 2022, pp. 373–384. | NO | No analysis by race/ethnicity |
| 51 | Doucette, Mitchell L., Alexander D. McCourt, Cassandra K. Crifasi, and Daniel W. Webster, “Impact of Changes to Concealed-Carry Weapons Laws on Fatal and Nonfatal Violent Crime, 1980–2019,” American Journal of Epidemiology, Vol. 192, No. 3, 2023, pp. 342–355. | NO | No analysis by race/ethnicity |
| 52 | Duggan M. More guns, more crime. *Journal of Political Economy*. 2001;109(5): 1086-1114 | NO | No analysis by race/ethnicity |
| 53 | Durlauf SN, Navarro S, Rivers DA. Model uncertainty and the effect of shall-issue right-to-carry laws on crime. *European Economic Review*. 2016;81: 32-67. | NO | No analysis by race/ethnicity |
| 54 | Duwe G, Kovandzic T, Moody CE. Impact of right-to-carry concealed firearm laws on mass public shootings. *Homicide Studies*. 2002;6(4): 271-296. | NO | No analysis by race/ethnicity |
| 55 | Edwards, Griffin, Erik Nesson, Joshua J. Robinson, and Fredrick Vars, “Looking Down the Barrel of a Loaded Gun: The Effect of Mandatory Handgun Purchase Delays on Homicide and Suicide,” Economic Journal, Vol. 128, No. 616, 2018, pp. 3117–3140. | YES |  |
| 56 | French B, Heagerty PJ. Analysis of longitudinal data to evaluate a policy change. *Stat Med*. 2008;27(24): 5005-25. | NO | No analysis by race/ethnicity |
| 57 | Fridel, Emma E., “Comparing the Impact of Household Gun Ownership and Concealed Carry Legislation on the Frequency of Mass Shootings and Firearms Homicide,” Justice Quarterly, Vol. 38, No. 5, 2021a, pp. 892–915. | NO | No analysis by race/ethnicity |
| 58 | Fridel, Emma E., “The Futility of Shooting Down Strawmen: A Response to Kleck (2020),” Justice Quarterly, Vol. 38, No. 5, 2021, pp. 925–941. | NO | No analysis by race/ethnicity |
| 59 | Ginwalla R, Rhee P, Friese R, Green DJ, Gries L, Joseph B, Kulvatunyou N, Lubin D, O'Keeffe T, Vercruysse G, Wynne J, Tang A. Repeal of the concealed weapons law and its impact on gun- related injuries and deaths. *Journal of Trauma and Acute Care Surgery*. 2014;76(3): 569-574 | NO | No analysis by race/ethnicity |
| 60 | Gius M. An examination of the effects of concealed weapons laws and assault weapons bans on state-level murder rates. *Applied Economics Letters*. 2014;21(4): 265-267. | NO | No analysis by race/ethnicity |
| 61 | Gius M. The Effects of State and Federal Background Checks on State-Level Gun-Related Murder Rates. Applied Economics. 2015;47(37-39): 4090-4101. | NO | No analysis by race/ethnicity |
| 62 | Gius M. The impact of minimum age and child access prevention laws on firearm-related youth suicides and unintentional deaths. *Social Science Journal*. 2015;52(2): 168-175. | NO | No analysis by race/ethnicity |
| 63 | Gius M. The impact of state and federal assault weapons bans on public mass shootings. *Applied Economics Letters*. 2015;22(4): 281-284. | NO | No analysis by race/ethnicity |
| 64 | Gius M. The Relationship Between Stand-Your-Ground Laws and Crime: A State-Level Analysis,” Social Science Journal, Vol. 53, No. 3, 2016, pp. 329–338. | NO | No analysis by race/ethnicity |
| 65 | Gius M. Effects of permit-to-purchase laws on state-level firearm murder rates. *Atlantic Economic Journal*. 2017;45(1): 73-80. | NO | No analysis by race/ethnicity |
| 66 | Gius M. The effects of state and Federal gun control laws on school shootings. *Applied Economics Letters*. 2018;25(5): 317-320. | NO | No analysis by race/ethnicity |
| 67 | Gius M. “Campus Crime and Concealed Carry Laws: Is Arming Students the Answer?” Social Science Journal, Vol. 56, No. 1, March 2019a, pp. 3–9. | NO | No analysis by race/ethnicity |
| 68 | Gius M. “Using the Synthetic Control Method to Determine the Effects of Concealed Carry Laws on State-Level Murder Rates,” International Review of Law and Economics, Vol. 57, March 2019b. | NO | No analysis by race/ethnicity |
| 69 | Gius M. “Examining the Impact of Child Access Prevention Laws on Youth Firearm Suicides Using the Synthetic Control Method,” International Review of Law and Economics, Vol. 63, September 2020a, 105914. | NO | No analysis by race/ethnicity |
| 70 | Gius M.“Using the Synthetic Control Method to Determine the Effects of Firearm Seizure Laws on State-Level Murder Rates,” Applied Economics Letters, Vol. 27, No. 21, 2020b, pp. 1754–1758. | NO | No analysis by race/ethnicity |
| 71 | Glaeser EL, Glendon S. Who owns guns? Criminals, victims, and the culture of violence. *American Economic Review*. 1998;88(2): 458-462 | NO | No analysis by race/ethnicity |
| 72 | Gobaud, Ariana N., Ahuva L. Jacobowitz, Christina A. Mehranbod, Nadav L. Sprague, Charles C. Branas, and Christopher N. Morrison, “Place-Based Interventions and the Epidemiology of Violence Prevention,” Current Epidemiology Reports, Vol. 9, No. 4, 2022, pp. 316–325. | NO | No analysis by race/ethnicity |
| 73 | Grambsch P. Regression to the Mean, Murder Rates, and Shall-Issue Laws. *American Statistician*. 2008;62(4): 289-295. | NO | No analysis by race/ethnicity |
| 74 | Guettabi M, Munasib A. Stand Your Ground laws, homicides and gun deaths. *Regional Studies*. 2018;52(9): 1250-1260. | NO | No analysis by race/ethnicity |
| 75 | Hamill ME, Hernandez MC, Bailey KR, Zielinski MD, Matos MA, Schiller HJ. State level firearm concealed carry legislation and rates of homicide and violent crime. *Journal of the American Academy of Surgeons.* 2018;228(1):1-8. | NO | No analysis by race/ethnicity |
| 76 | Hamlin, Daniel, “Are Gun Ownership Rates and Regulations Associated with Firearm Incidents in American Schools? A Forty-Year Analysis (1980–2019),” Journal of Criminal Justice, Vol. 76, 2021, 101847. | NO | No analysis by race/ethnicity |
| 77 | Hasegawa, Raiden B., Daniel W. Webster, and Dylan S. Small, “Evaluating Missouri’s Handgun Purchaser Law: A Bracketing Method for Addressing Concerns About History Interacting with Group,” Epidemiology, Vol. 30, No. 3, May 2019, pp. 371–379. | NO | No analysis by race/ethnicity |
| 78 | Helland E, Tabarrok A. Using placebo laws to test "more guns, less crime." *Advances in Economic Analysis & Policy*. 2004;4(1): 1-7 | NO | No analysis by race/ethnicity |
| 79 | Hempstead K, Rodríguez-Andrés A. *Gun control and suicide: the impact of state firearm regulations, 1995 - 2004*. Development research working paper series; 2009. | NO | No analysis by race/ethnicity |
| 80 | Hepburn, Lisa, Matthew Miller, Deborah Azrael, and David Hemenway, “The Effect of Nondiscretionary Concealed Weapon Carrying Laws on Homicide,” Journal of Trauma: Injury, Infection, and Critical Care, Vol. 56, No. 3, 2004, pp. 676–681. | YES |  |
| 81 | Hepburn L, Azrael D, Miller M, Hemenway D. The effect of child access prevention laws on unintentional child firearm fatalities, 1979-2000. *Journal of Trauma-Injury Infection and Critical Care*. 2006;61(2): 423-428. | NO | No analysis by race/ethnicity |
| 82 | Humphreys DK, Gasparrini A, Wiebe DJ. Evaluating the impact of Florida's "stand your ground" self-defense law on homicide and suicide by firearm: An interrupted time series study. *JAMA Int Med*. 2017;177(1): 44-50. | NO | No analysis by race/ethnicity that meets inclusion criteria |
| 83 | Kagawa RMC, Castillo-Carniglia A, Vernick JS, Webster D, Crifasi C, Rudolph KE, Cerdá M, Shev A, Wintemute GJ. Repeal of comprehensive background check policies and firearm homicide and suicide. *Epidemiology*. 2018;29(4): 494-502. | NO | No analysis by race/ethnicity |
| 84 | Kagawa, Rose, Amanda Charbonneau, Christopher McCort, Alexander McCourt, Jon Vernick, Daniel Webster, and Garen Wintemute, “Effects of Comprehensive Background Check Policies on Firearm Fatalities in Four States,” American Journal of Epidemiology, 2023. | NO | No analysis by race/ethnicity |
| 85 | Kalesan B, Lagast K, Villarreal M, Pino E, Fagan J, Galea S. School shootings during 2013-2015 in the USA. *Inj Prev*. 2017;23(5): 321-327. | NO | No analysis by race/ethnicity |
| 86 | Kappelman, Jack, and Richard C. Fording, “The Effect of State Gun Laws on Youth Suicide by Firearm: 1981–2017,” Suicide and Life‐Threatening Behavior, Vol. 51, No. 2, April 2021, pp. 368–377. | NO | No analysis by race/ethnicity |
| 87 | Kaufman, Elinore J., Christopher N. Morrison, Erik J. Olson, David K. Humphreys, Douglas J. Wiebe, Niels D. Martin, Carrie A. Sims, Mark H. Hoofnagle, C. William Schwab, Patrick M. Reilly, and Mark J. Seamon, “Universal Background Checks for Handgun Purchases Can Reduce Homicide Rates of African Americans,” Journal of Trauma and Acute Care Surgery, Vol. 88, No. 6, June 2020, pp. 825–831. | YES |  |
| 88 | Kendall, Todd D., and Robert Tamura, “Unmarried Fertility, Crime, and Social Stigma,” Journal of Law and Economics, Vol. 53, No. 1, 2010, pp. 185–221. | NO | No analysis by race/ethnicity |
| 89 | Kester, Louis, Daniel N. Holena, Allyson M. Hynes, Elinore J. Kaufman, Tejal Brahmbhatt, Sabrina Sanchez, James P. Byrne, Tracey Dechert, Mark Seamon, and Dane R. Scantling, “Preventing the Most Common Firearm Deaths: Modifiable Factors Related to Firearm Suicide,” Surgery, Vol. 173, No. 2, 2023, pp. 544–552. | NO | No analysis by race/ethnicity |
| 90 | Kivisto, Aaron J., Katherine L. Kivisto, Erica Gurnell, Peter Phalen, and Bradley Ray, “Adolescent Suicide, Household Firearm Ownership, and the Effects of Child Access Prevention Laws,” Journal of the American Academy of Child and Adolescent Psychiatry, Vol. 60, No. 9, September 2021, pp. 1096–1104. | NO | No analysis by race/ethnicity |
| 91 | Kivisto AJ, Phalen PL. Effects of Risk-Based Firearm Seizure Laws in Connecticut and Indiana on Suicide Rates, 1981-2015. *Psychiatr Serv*. 2018;69(8): 855-862 | NO | No analysis by race/ethnicity |
| 92 | Klarevas, Louis, Andrew Conner, and David Hemenway, “The Effect of Large-Capacity Magazine Bans on High-Fatality Mass Shootings, 1990–2017,” American Journal of Public Health, Vol. 109, No. 12, 2019, pp. 1754–1761. | NO | No analysis by race/ethnicity |
| 93 | Knopov, Anita, Michael Siegel, Ziming Xuan, Emily F. Rothman, Shea W. Cronin, and David Hemenway, “The Impact of State Firearm Laws on Homicide Rates Among Black and White Populations in the United States, 1991–2016,” Health and Social Work, Vol. 44, No. 4, 2019, pp. 232–240. | YES |  |
| 94 | Koper CS, Roth JA. The impact of the 1994 Federal Assault Weapons Ban on gun markets: An assessment of short-term primary and secondary market effects. *Journal of Quantitative Criminology*. 2002;18(3): 239-266 | NO | No analysis by race/ethnicity |
| 95 | Koper CS. *Updated assessment of the federal assault weapon ban: Impacts of gun markets & gun violence, 1994-2003*. National Institute of Justice, Washington, DC; 2004. | NO | No analysis by race/ethnicity |
| 96 | Koper CS, Roth JA. The impact of the 1994 Federal Assault Weapon Ban on gun violence outcomes: An assessment of multiple outcome measures and some lessons for policy evaluation. *Journal of Quantitative Criminology*. 2001;17(1): 33 | NO | No analysis by race/ethnicity |
| 97 | Koper CS, Roth JA. The impact of the 1994 Federal Assault Weapons Ban on gun markets: An assessment of short-term primary and secondary market effects. *Journal of Quantitative Criminology*. 2002;18(3): 239-266 | NO | No analysis by race/ethnicity |
| 98 | Kovandzic TV, Marvell TB, Vieraitis LM. The impact of "shall-issue" concealed handgun laws on violent crime rates - Evidence from panel data for large urban cities. *Homicide Studies*. 2005;9(4): 292-323. | NO | No analysis by race/ethnicity |
| 99 | La Valle JM. Rebuilding at gunpoint: A city-level re-estimation of the Brady Law and RTC laws in the wake of Hurricane Katrina. *Criminal Justice Policy Review*. 2007;18(4): 451-465. | NO | No analysis by race/ethnicity |
| 100 | La Valle JM. Re-estimating gun-policy effects according to a National Science Academy Report: Were previous reports of failure pre-mature? *Journal of Crime & Justice*. 2010;33(1): 71-95. | NO | No analysis by race/ethnicity |
| 101 | La Valle JM. Gun control” vs.“self-protection”: A case against the ideological divide. *Justice Policy Journal.* 2013;10(1):1-26. | NO | No analysis by race/ethnicity |
| 102 | La Valle JM, Glover TC. Revisiting Licensed Handgun Carrying: Personal Protection or Interpersonal Liability? *American Journal of Criminal Justice.* 2012;37(4):580-601. | NO | No analysis by race/ethnicity |
| 103 | Levy, Marc, Wilmer Alvarez, Lauren Vagelakos, Michelle Yore, and Bertha Ben Khallouq, “Stand Your Ground: Policy and Trends in Firearm-Related Justifiable Homicide and Homicide in the US,” Journal of the American College of Surgeons, Vol. 230, No. 1, January 2020, pp. 161–167. | NO | No analysis by race/ethnicity |
| 104 | Liu, Ye, Michael Siegel, and Bisakha Sen, “Association of State-Level Firearm-Related Deaths with Firearm Laws in Neighboring States,” JAMA Network Open, Vol. 5, No. 11, 2022, pp. e2240750–e2240750. | NO | No analysis by race/ethnicity |
| 105 | Lott Jr JR. The concealed-handgun debate. *Journal of Legal Studies*. 1998;27(1): 221 | NO | No analysis by race/ethnicity |
| 106 | Lott Jr, JR. *More Guns, Less Crime: Understanding Crime and Gun Control Laws*, 1st ed., University of Chicago Press, Chicago, IL; 1998. | NO | No analysis by race/ethnicity |
| 107 | Lott Jr JR. *More Guns, Less Crime: Understanding Crime and Gun-Control Laws*, 2nd ed. University of Chicago Press, Chicago, IL; 2000. | NO | No analysis by race/ethnicity |
| 108 | Lott Jr JR. *Bias against guns: Why almost everything you've heard about gun control is wrong*. Regnery Publishing, Inc., Washington, DC; 2003. | NO | No analysis by race/ethnicity |
| 109 | Lott Jr JR. *More Guns, Less Crime: Understanding Crime and Gun-Control Laws*, 3rd ed., University of Chicago Press, Chicago, IL; 2010. | NO | No analysis by race/ethnicity |
| 110 | Lott, John R., Jr., and William M. Landes, Multiple Victim Public Shootings, Bombings, and Right-to-Carry Concealed Handgun Laws: Contrasting Private and Public Law Enforcement, Chicago, Ill.: University of Chicago Law School, John M. Olin Law and Economics Working Paper No. 73, 1999. | NO | No analysis by race/ethnicity |
| 111 | Lott, John R., Jr., and D. B. Mustard, “Crime, Deterrence, and Right-to-Carry Concealed Handguns,” Journal of Legal Studies, Vol. 26, No. 1, 1997, pp. 1–68. | YES |  |
| 112 | Lott Jr JR, Whitley JE. Safe-storage gun laws: Accidental deaths, suicides, and crime. *Journal of Law and Economics*. 2001;44(3): 659-689 | NO | No analysis by race/ethnicity |
| 113 | Lott Jr JR, Whitley J. Measurement error in county-level UCR data. *Journal of Quantitative Criminology*. 2003;19: 185-198. | NO | No analysis by race/ethnicity |
| 114 | Lott Jr JR, Whitley JE. Abortion and crime: Unwanted children and out-of-wedlock births. *Economic Inquiry*. 2007;45(2): 304-324. | NO | No analysis by race/ethnicity |
| 115 | Luca M, Malhotra D, Poliquin C. *The Impact of Mass Shootings on Gun Policy*. Harvard Business School Working Paper; 2016. | NO | No analysis by race/ethnicity |
| 116 | Luca M, Malhotra D, Poliquin C. Handgun waiting periods reduce gun deaths. *P Natl Acad Sci USA.* 2017;114(46):12162-12165. | NO | No analysis by race/ethnicity |
| 117 | Luca M, Malhotra D, Poliquin C. “The Impact of Mass Shootings on Gun Policy,” Journal of Public Economics, Vol. 181, 2020, 104083. | NO | No analysis by race/ethnicity |
| 118 | Ludwig J. Concealed-gun-carrying laws and violent crime: Evidence from state panel data. *International Review of Law & Economics*. 1998;18(3): 239-254 | NO | No analysis by race/ethnicity |
| 119 | Ludwig J, Cook PJ. Homicide and suicide rates associated with implementation of the Brady Handgun Violence Prevention Act. *J Am Med Assoc.* 2000;284(5):585-591. | NO | No analysis by race/ethnicity |
| 120 | Manski CF, Pepper JV. *How Do Right-To-Carry Laws Affect Crime Rates? Coping with Ambiguity Using Bounded-Variation Assumptions*. NBER Working Paper; 2015. | NO | No analysis by race/ethnicity |
| 121 | Manski CF, Pepper JV. How do right-to-carry laws affect crime rates? Coping with ambiguity using bounded-variation assumptions. *Review of Economics and Statistics*. 2018;100(2): 232-244. | NO | No analysis by race/ethnicity |
| 122 | Martin RA, Legault RL. Systematic measurement error with state-level crime data: Evidence from the "more guns, less crime" debate. *Journal of Research in Crime and Delinquency*. 2005;42(2): 187-210. | NO | No analysis by race/ethnicity |
| 123 | Marvell TB. The impact of banning juvenile gun possession. *The Journal of Law and Economics.* 2001;44(S2):691-713. | NO | No analysis by race/ethnicity |
| 124 | McClellan, Chandler, and Erdal Tekin, “Stand Your Ground Laws, Homicides, and Injuries,” Journal of Human Resources, Vol. 52, No. 3, 2017, pp. 621–653. | YES |  |
| 125 | McCourt, Alexander D., Cassandra K. Crifasi, Elizabeth A. Stuart, Jon S. Vernick, Rose M. C. Kagawa, Garen J. Wintemute, and Daniel W. Webster, “Purchaser Licensing, Point-of-Sale Background Check Laws, and Firearm Homicide and Suicide in 4 US States, 1985–2017,” American Journal of Public Health, Vol. 110, No. 10, October 2020, pp. 1546–1552. | NO | No analysis by race/ethnicity |
| 126 | Miller, Megan, and John Pepper, “Assessing the Effect of Firearms Regulations Using Partial Identification Methods: A Case Study of the Impact of Stand Your Ground Laws on Violent Crime,” Law and Contemporary Problems, Vol. 83, 2020, pp. 213–230. | NO | No analysis by race/ethnicity |
| 127 | Moe, Caitlin A., Miriam J. Haviland, Andrew G. Bowen, Ali Rowhani-Rahbar, and Frederick P. Rivara, “Association of Minimum Age Laws for Handgun Purchase and Possession with Homicides Perpetrated by Young Adults Aged 18 to 20 Years,” JAMA Pediatrics, Vol. 174, No. 11, 2020, pp. 1056–1062. | NO | No analysis by race/ethnicity |
| 128 | Monroe JD. *Homicide and gun control: the Brady Handgun Violence Prevention Act and homicide rates*. LFB Scholarly Publishing LLC; 2008. | NO | No analysis by race/ethnicity |
| 129 | Moody CE. Testing for the effects of concealed weapons laws: Specification errors and robustness. *Journal of Law & Economics*. 2001;44(2): 799-813 | NO | No analysis by race/ethnicity |
| 130 | Moody CE, Marvell TB. The debate on shall-issue laws. *Econ Journal Watch*. 2008;5(3): 269-293 | NO | No analysis by race/ethnicity |
| 131 | Moody C, Marvell TB. The debate on shall issue laws, continued. *Econ Journal Watch*. 2009;6(2): 203-217 | NO | No analysis by race/ethnicity |
| 132 | Moody CE, Marvell TB. Clustering and standard error bias in fixed effects panel data regressions. *J Quant Criminol*; 2018. | NO | No analysis by race/ethnicity |
| 133 | Moody CE, Marvell TB. The impact of right-to-carry laws: A critique of the 2014 version of Aneja, Donohue, and Zhang. *Econ Journal Watch*. 2018;15(1). | NO | No analysis by race/ethnicity |
| 134 | Moody CE, Marvell TB, Zimmerman PR, Alemante F. The impact of right-to-carry laws on crime: An exercise in replication. *Review of Economics and Finance*. 2014;4: 33-43. | NO | No analysis by race/ethnicity |
| 135 | Munasib A, Kostandini G, Jordan JL. Impact of the Stand Your Ground law on gun deaths: Evidence of a rural urban dichotomy. *European Journal of Law and Economics*. 2018;45(3): 527-554. | NO | No analysis by race/ethnicity |
| 136 | Mustard DB. The impact of gun laws on police deaths. *Journal of Law and Economics*. 2001;44(3): 635-657 | NO | No analysis by race/ethnicity |
| 137 | Neufeld, Miriam Y., Michael Poulson, Sabrina E. Sanchez, and Michael B. Siegel, “State Firearm Laws and Nonfatal Firearm Injury-Related Inpatient Hospitalizations: A Nationwide Panel Study,” Journal of Trauma and Acute Care Surgery, Vol. 92, No. 3, 2022, pp. 581–587. | NO | No analysis by race/ethnicity |
| 138 | Oliphant, Stephen N., “Effects of Wisconsin’s Handgun Waiting Period Repeal on Suicide Rates,” Injury Prevention, Vol. 28, No. 6, 2022, pp. 580–584. | NO | No analysis by race/ethnicity |
| 139 | Olson DE, Maltz MD. Right-to-carry concealed weapon laws and homicide in large U.S. counties: The effect on weapon types, victim characteristics, and victim-offender relationships. *J Law Econ*. 2001;44, 747. | YES |  |
| 140 | Pear, Veronica A., Garen J. Wintemute, Nicholas P. Jewell, and Jennifer Ahern, “Firearm Violence Following the Implementation of California’s Gun Violence Restraining Order Law,” JAMA Network Open, Vol. 5, No. 4, 2022, pp. e224216–e224216. | YES |  |
| 141 | Plassmann F, Tideman T. Does the right to carry concealed handguns deter countable crimes? Only a count analysis can say. *Journal of Law and Economics*. 2001;44(3): 771-798 | NO | No analysis by race/ethnicity |
| 142 | Plassman F, Whitley J. Confirming More Guns, Less Crime. *Stanford Law Review*. 2003;55(4): 1313-1369. | NO | No analysis by race/ethnicity |
| 143 | Raifman, Julia, Elysia Larson, Colleen L. Barry, Michael Siegel, Michael Ulrich, Anita Knopov, and Sandro Galea, “State Handgun Purchase Age Minimums in the US and Adolescent Suicide Rates: Regression Discontinuity and Difference-in-Differences Analyses,” BMJ, Vol. 370, 2020. | NO | No analysis by race/ethnicity |
| 144 | Raissian KM. Hold your fire: Did the 1996 Federal Gun Control Act expansion reduce domestic homicides? *Journal of Policy Analysis and Management*. 2016;35(1): 67. | NO | No analysis by race/ethnicity |
| 145 | Roberts DW. Intimate partner homicide: Relationships to alcohol and firearms. *Journal of Contemporary Criminal Justice*. 2009;25(1): 67-88. | NO | No analysis by race/ethnicity |
| 146 | Rochford, Hannah I., Mark Berg, and Corinne Peek-Asa, “The ‘Boyfriend Loophole’ and Intimate Partner Homicides: A Longitudinal Analysis Using the National Violent Death Reporting System,” Journal of Prevention, 2022, pp. 1–19. | YES |  |
| 147 | Rogna, Marco, and Bich Diep Nguyen, “Firearms Law and Fatal Police Shootings: A Panel Data Analysis,” Applied Economics, Vol. 54, No. 27, 2022, pp. 3121–3137. | NO | No analysis by race/ethnicity |
| 148 | Rosengart M, Cummings P, Nathens A, Heagerty P, Maier R, Rivara F. An evaluation of state firearm regulations and homicide and suicide death rates. *Inj Prev.* 2005;11(2):77-83. | NO | No analysis by race/ethnicity |
| 149 | Roth JA, Koper CS, Adams W. *Impact evaluation of the Public Safety and Recreational Firearms Use Protection Act of 1994: final report*. National Institute of Justice, Washington, DC; 1997. | NO | No analysis by race/ethnicity |
| 150 | Roth JA, Koper CS. *Impact of the 1994 Assault Weapons Ban: 1994-96, Research in Brief*. National Institute of Justice, Washington, DC; 1999. | NO | No analysis by race/ethnicity |
| 151 | Rubin, Paul H., and Hashem Dezhbakhsh, “The Effect of Concealed Handgun Laws on Crime: Beyond the Dummy Variables,” International Review of Law and Economics, Vol. 23, No. 2, 2003, pp. 199–216. | YES |  |
| 152 | Rudolph KE, Stuart EA, Vernick JS, Webster DW. Association between Connecticut's permit-to-purchase handgun law and homicides. *Am J Public Health*. 2015;105(8): e49-54. | NO | No analysis by race/ethnicity |
| 153 | Saadi, Altaf, Kristen R. Choi, Sae Takada, and Fred J. Zimmerman, “The Impact of Gun Violence Restraining Order Laws in the U.S. and Firearm Suicide Among Older Adults: A Longitudinal State-Level Analysis, 2012–2016,” BMC Public Health, Vol. 20, No. 334, 2020. | NO | No analysis by race/ethnicity |
| 154 | Sabbath, Erika L., Summer Sherburne Hawkins, and Christopher F. Baum, “State-Level Changes in Firearm Laws and Workplace Homicide Rates: United States, 2011 to 2017,” American Journal of Public Health, Vol. 110, No. 2, 2020, pp. 230–236. | NO | No analysis by race/ethnicity |
| 155 | Scantling, Dane R., Daniel N. Holena, Elinore J. Kaufman, Allyson M. Hynes, Justin Hatchimonji, James P. Byrne, Douglas Wiebe, and Mark J. Seamon, “Modifiable Factors Related to Firearm Homicides: A Broader View of Our Lane,” Annals of Surgery, 2022. | NO | No analysis by race/ethnicity |
| 156 | Schell, Terry L., Matthew Cefalu, Beth Ann Griffin, Rosanna Smart, and Andrew R. Morral, “Changes in Firearm Mortality Following the Implementation of State Laws Regulating Firearm Access and Use,” Proceedings of the National Academy of Sciences, Vol. 117, No. 26, 2020, pp. 14906–14910. | NO | No analysis by race/ethnicity |
| 157 | Sen B, Panjamapirom A. State background checks for gun purchase and firearm deaths: an exploratory study. *Prev Med.* 2012;55(4):346-350. | NO | No analysis by race/ethnicity |
| 158 | Shi W, Lee L. The effects of gun control on crimes: a spatial interactive fixed effects approach. *Empirical Economics*. 2018;55(1): 233-263. | NO | No analysis by race/ethnicity |
| 159 | Siegel M, Xuan Z, Ross CS, et al. Easiness of legal access to concealed firearm permits and homicide rates in the United States. *Am J Public Health.* 2017;107(12):1923-1929. | NO | No analysis by race/ethnicity |
| 160 | Siegel, Michael, Molly Pahn, Ziming Xuan, Eric Fleegler, and David Hemenway, “The Impact of State Firearm Laws on Homicide and Suicide Deaths in the USA, 1991–2016: A Panel Study,” Journal of General Internal Medicine, Vol. 34, No. 10, 2019, pp. 2021–2028. | NO | No analysis by race/ethnicity |
| 161 | Siegel, Michael, Max Goder-Reiser, Grant Duwe, Michael Rocque, James Alan Fox, and Emma E. Fridel, “The Relation Between State Gun Laws and the Incidence and Severity of Mass Public Shootings in the United States, 1976–2018,” Law and Human Behavior, Vol. 44, No. 5, 2020, 347. | NO | No analysis by race/ethnicity |
| 162 | Siegel, Michael, Benjamin Solomon, Anita Knopov, Emily F. Rothman, Shea W. Cronin, Ziming Xuan, and David Hemenway, “The Impact of State Firearm Laws on Homicide Rates in Suburban and Rural Areas Compared to Large Cities in the United States, 1991–2016,” Journal of Rural Health, Vol. 36, No. 2, 2020b, pp. 255–265. | NO | No analysis by race/ethnicity |
| 163 | Smith, Michael R., and Matthew Petrocelli, “The Effect of Concealed Handgun Carry Deregulation in Arizona on Crime in Tucson,” Criminal Justice Policy Review, Vol. 30, No. 8, 2019, pp. 1186–1203. | NO | No analysis by race/ethnicity |
| 164 | Steidley, Trent, “The Effect of Concealed Carry Weapons Laws on Firearm Sales,” Social Science Research, Vol. 78, February 2019. | NO | No analysis by race/ethnicity |
| 165 | Steidley T, Kosla MT. Toward a Status Anxiety Theory of Macro-level Firearm Demand. *Social Currents*. 2018;5(1): 86-103. | NO | No analysis by race/ethnicity |
| 166 | Strnad J. Should legal empiricists go Bayesian? *American Law and Economics Review*. 2007;9(1): 195-303. | NO | No analysis by race/ethnicity |
| 167 | Swanson, J. W., A. G. Robertson, L. K. Frisman, M. A. Norko, H. Lin, M. S. Swartz, and P. J. Cook, “Preventing Gun Violence Involving People with Serious Mental Illness,” in D. W. Webster and J. S. Vernick, eds., Reducing Gun Violence in America: Informing Policy with Evidence and Analysis, Baltimore, Md.: Johns Hopkins University Press, 2013, pp. 33–51. | NO | No analysis by race/ethnicity |
| 168 | Swanson JW, Easter MM, Robertson AG, Swartz MS, Alanis-Hirsch K, Moseley D, Dion C, Petrila J. Gun violence, mental illness, and laws that prohibit gun possession: Evidence from two Florida counties. *Health Aff (Millwood).* 2016;35(6): 1067-75. | NO | No analysis by race/ethnicity |
| 169 | Van Der Wal, Willem M., “Marginal Structural Models to Estimate Causal Effects of Right-to-Carry Laws on Crime,” Statistics and Public Policy, Vol. 9, No. 1, 2022, pp. 163–174. | NO | No analysis by race/ethnicity |
| 170 | Vigdor ER, Mercy JA. Disarming Batters: The Impact of Domestic Violence Firearm Laws [From Evaluating Gun Policy: Effects on Crime and Violence, P 157-214, 2003, Philip J. Cook and Jens Ludwig, eds]; 2003. | NO | No analysis by race/ethnicity |
| 171 | Vigdor ER, Mercy JA. Do laws restricting access to firearms by domestic violence offenders prevent intimate partner homicide? *Eval Rev*. 2006;30(3): 313-346. | NO | No analysis by race/ethnicity |
| 172 | Wallace LN. Castle Doctrine legislation: Unintended effects for gun ownership? *Justice Policy Journal*. 2014;11(2). | NO | No analysis by race/ethnicity |
| 173 | Wallace, Maeve E., Dovile Vilda, Katherine P. Theall, and Charles Stoecker, “Firearm Relinquishment Laws Associated with Substantial Reduction in Homicide of Pregnant and Postpartum Women,” Health Affairs, Vol. 40, No. 10, 2021, pp. 1654–1662. | NO | No analysis by race/ethnicity |
| 174 | Wallin, Mikaela A., Charvonne N. Holliday, and April M. Zeoli, “The Association of Federal and State-Level Firearm Restriction Policies with Intimate Partner Homicide: A Re-Analysis by Race of the Victim,” Journal of Interpersonal Violence, Vol. 37, No. 17-18, 2022, NP16509–NP16533. | YES |  |
| 175 | Webster D, Crifasi CK, Vernick JS. Effects of the repeal of Missouri’s handgun purchaser licensing law on homicides. *J Urban Health.* 2014;91(2):293-302. | NO | No analysis by race/ethnicity |
| 176 | Webster DW, Vernick JS, Zeoli AM, Manganello JA. Association between youth-focused firearm laws and youth suicides. *JAMA.* 2004;292(5):594-601. | NO | No analysis by race/ethnicity |
| 177 | Webster, Daniel W., Alexander D. McCourt, Cassandra K. Crifasi, Marisa D. Booty, and Elizabeth A. Stuart, “Evidence Concerning the Regulation of Firearms Design, Sale, and Carrying on Fatal Mass Shootings in the United States,” Criminology and Public Policy, Vol. 19, No. 1, 2020, pp. 171–212. | NO | No analysis by race/ethnicity |
| 178 | Webster DW, Starnes M. Reexamining the association between child access prevention gun laws and unintentional shooting deaths of children. *Pediatrics*. 2000;106(6): 1466-9 | NO | No analysis by race/ethnicity |
| 179 | Webster DW, Vernick JS, Hepburn LM. Effects of Maryland's law banning "Saturday night special" handguns on homicides. *Am J Epidemiol*. 2002;155(5): 406-12 | NO | No analysis by race/ethnicity |
| 180 | Zeoli AM, McCourt A, Buggs S, Frattaroli S, Lilley D, Webster DW. Analysis of the strength of legal firearms restrictions for perpetrators of domestic violence and their associations with intimate partner homicide. *Am J Epidemiology*. 2018;187(7): 1449-1455. | NO | No analysis by race/ethnicity |
| 181 | Zeoli AM, Webster DW. Effects of domestic violence policies, alcohol taxes and police staffing levels on intimate partner homicide in large US cities. *Inj Prev*. 2010;16(2): 90-95. | NO | No analysis by race/ethnicity |
| 182 | Zimmerman, Paul R., “The Deterrence of Crime Through Private Security Efforts: Theory and Evidence,” International Review of Law and Economics, Vol. 37, 2014, pp. 66–75. | NO | No analysis by race/ethnicity |

**Appendix B: Full search string for Scopus Updated Search**

See Smart et al. (2023) for information on the preceding search of studies published between 1994 through October 2020.

**Table B.1. Full Scopus Search String**

| **Database (Search execution date)** | **Search String** | **Year Limiter** | **Other Limiters** |
| --- | --- | --- | --- |
| Scopus  (March 1, 2023) | TITLE- ABSTRACT- KEYWORD ( gun OR guns OR firearm* OR handgun* OR shotgun* OR rifle* OR longgun or machinegun* OR pistol* OR "automatic weapon*" OR "assault weapon*" OR "semi-automatic weapon*" OR "semiautomatic weapon*" OR "Saturday night special*")  **AND**  TITLE- ABSTRACT- KEYWORD ( ownership OR own OR owns OR availab* OR access* OR possess* OR purcha* OR restrict* OR regulat* OR distribut* OR "weapon carrying" OR "weapon- carrying" OR legislation OR legislating OR legislative OR law OR laws OR legal* OR policy OR policies OR ban OR bans OR banned OR "concealed carry" )  **AND**  TITLE- ABSTRACT- KEYWORD ( suicid* OR "self-harm*" OR "self-injur*" OR "self injur*" OR homicide* OR murder* OR manslaughter OR "domestic violence" OR "spousal abuse" OR "spouse abuse" OR "elder abuse" OR "child abuse" OR "family violence" OR "child maltreatment" OR "spousal maltreatment" OR "elder maltreatment" OR "elder mistreatment" OR "intimate relationship violence" OR "intimate partner violence" OR "dating violence" OR rape OR rapes OR rapist* OR "personal crime" OR "personal crimes" OR robbery OR robberies OR assault* OR stalk* OR terroris* OR (violen* AND (crime* OR criminal*)) OR accident* OR unintentional* OR "un intentional*" OR "mass shooting*" OR "school shooting*" OR "law enforcement" OR police* OR policing OR "self-defense" OR "self defense" OR "personal defense" OR defens* OR "self-protect*" OR "self protect*" OR DGU OR SDGU OR hunt OR hunting OR "sport shooting*" OR "shooting sports" OR recreation* OR industr* OR manufactur* OR produc* OR distribut* OR supply OR supplies OR trade* OR trading OR price* OR export* OR revenue* OR sales OR employ* OR profit* OR cost OR costs OR costing OR "gun show*" OR tax OR taxes OR taxing OR taxation OR payroll OR "federal firearms license*" OR "federal firearm license*" OR FFL) | 2019<PUBYEAR | LANGUAGE - English |

**Appendix C: Details on Quality Assessment Domains**

Table C.1 documents the quality assessment criteria used to assess potential methodological concerns with study effect estimates (defined at the study-policy-outcome) level. Ratings and guidance for determining ratings are shown by assessment domain.

Table C.2. documents how overall quality judgments for a given estimate are determined based on integrating across those domains shown in Table C.1.

Table C.3. maps our quality assessment domains to those in ROBINS-I and provides some narrative discussion of differences.

**Table C.1. Domain-specific judgments for a specific policy x outcome**

| Proposed domain | Definition | Rating (done at the policy x outcome level) |
| --- | --- | --- |
| **Threats to causal identification** | The extent to which a study design is likely to produce biased estimates of the causal effects of the policies of interest (i.e., a pre-post & comparison group approach is used, but there are concerns with interpreting the estimated policy effect as causal due to potential violation(s) of the model’s identifying assumptions). Note that policy estimates that are presented but are intended to represent covariate controls should be coded as serious or critical concerns (see Hünermund & Louw, 2022). | No concern (low risk of bias): Discusses the model’s identifying assumptions and provides evidence to support that assumptions are met or provides evidence of similar results under various reasonable assumptions.  Minor concern (moderate risk of bias): Does not discuss or provide evidence in support of models’ identifying assumption and/or validity of control group, but the approach does not have serious or critical concerns defined below.  Serious concern (serious risk of bias): Accounts for unit-level and time-level confounds, but:  (a) For regression-based approaches: does not account for any unit-specific time-varying confounds that are likely to affect both probability of policy adoption and the outcome of interest, *and* does not provide evidence in support of parallel trends or conditional ignorability assumptions (as relevant).  (b) For synthetic control methods: does not discuss or provide evidence of pre-period fit.  (c) For other less commonly used approaches, this will be rated via group discussion.  *or*  Accounts for unit-level, time-level, and unit-specific time-varying confounds (or lagged outcomes), but provides some evidence that identifying assumptions may be violated and does not address the violations.  *or*  Accounts for unit-level, time-level, and unit-specific time-varying confounds (or lagged outcomes), but the policy is not the focus of the paper (e.g., the policy is a control variable) and evidence specifically in support of meeting identifying assumptions for the ancillary policy are not discussed or provided.  Critical concern (critical risk of bias): Accounts for only unit-level or only time-level confounds (but not both, nor unit-specific time-varying confounds), and does not provide any justification as related to identifying assumptions.  *or*  Accounts for unit-level and time-level confounds, but provides strong evidence that identifying assumptions may be violated and does not adequately address the violations. |
| **Small number of treated units** | The extent to which a small number of treated units precludes the analyses’ ability to account for co-interventions or other changes that may have coincided with implementation of the policy of interest; note this issue further creates concerns with external generalizability and accurate inferential statistics. | No concern (low risk of bias): Four or more groups with policy transitions (e.g., implement law, repeal law) with 3+ years pre- and 3+ years post-transition data.  Minor concern (moderate risk of bias): Does not provide sufficient information on policy data source, coding method, or policy variation to assess the number of states contributing to identifying variation with pre-post data, but likely to meet criteria based on RAND legal database or State Firearm Law Database.  Serious concern (serious risk of bias): <4 treated groups with 3 years pre/post, *or* insufficient info and unlikely to meet criteria based on RAND legal database or State Firearm Law Database.  Critical concern (critical risk of bias): <4 treated groups with 3+ years pre and post policy data *and* <4 control units (or in SCM studies, >95% of weight drawn from 3 or fewer donors). |
| **Policy classification and coding** | Noted critical errors in policy classification. | No concern (low risk of bias): Assumed to hold unless literature has established critical errors in law coding.  Critical concern (critical risk of bias): Literature has established critical errors in law coding that are not explained or justified by the article. |
| **Missing data** | The extent to which missing data are likely to bias results. | No concern (low risk of bias): Data reasonably complete *or* proportions of and reasons for missing data similar across treated and control groups thus missing data unlikely to be correlated with policy *or* missing data are addressed appropriately.  Minor concern (moderate risk of bias): Substantial amount of missingness *or* proportions of and reasons for missing data differ slightly across treated and control groups (and not addressed analytically), but missing data unlikely to be correlated with policy.  Serious concern (serious risk of bias): Analyses omits outcome data that are related to outcome data values (e.g., dropping suppressed state-years from CDC WONDER; log-linear models that drop outcomes with value zero) but this affects a reasonably small proportion of observations. Do not treat this as a serious issue if states/units with suppression are dropped in their entirety (rather than dropping unit-years)  Critical concern (critical risk of bias): Omission of outcome data that are related to outcome data values (e.g., dropping suppressed state-years from CDC WONDER; log-linear models that drop outcomes with value zero) and affects a high proportion of observations. |
| **Violation of model assumptions & inferential statistics concerns** |  | No concern (low risk of bias): No serious concerns with distributional assumptions; methods correct for non-independence (e.g., clustered SEs)  Minor concern (moderate risk of bias): No serious concerns with distributional assumptions or serial correlation, but potential concerns due to some other forms of non-independence (e.g., meta-analyzing state-specific estimates from SCM) not adequately addressed.  Serious concern (serious risk of bias): Serial correlation not addressed (for outcomes likely to be characterized by strong serial correlation) *or* linear models of logged outcomes where outcomes are rare enough such that there are a nontrivial number of zero cells *or* linear models of binary outcomes with prevalence <15% (or higher than 85%).  *and*  no additional specifications or evidence are provided to support validity of distributional assumptions or effect estimates.  Critical concern (critical risk of bias): Inferential statistics for relevant policy effect estimates are not provided or are estimated in a way that is critically inappropriate given the approach (e.g., use of basic t-tests with synthetic control methods). |
| **Potential for model overfitting** | The extent to which issues related to potential model overfit are likely to threaten the validity of the estimated effects and inferential statistics, with no supplemental information provided to reduce concerns. This will be assessed by calculating observations per parameter (n:k) and events per parameter (e:k). | No concern (low risk of bias): n:k>=10 *and* e:k>=10.  Minor concern (moderate risk of bias): (5< n:k < 10 & 10=<e:k) *or* (10<=n:k & 5<e:k<10).  Serious concern (serious risk of bias): 5<n:k<10 *and* 5<e:k<10.    Critical concern (critical risk of bias): n:k<5 or e:k <5. |
| **Other** | Judged on a case-by-case basis. | Judged on a case-by-case basis. |

**Table C.2. Overall quality judgment for a specific policy x outcome (across domains)**

| Response option | Criteria |
| --- | --- |
| High methodological quality | No issues for all domains |
| High methodological quality with minor concerns | No or minor methodological concerns across all domains |
| Serious methodological concerns | Serious methodological concerns in at least one domain, but no critical methodological concerns in any domain |
| Critical methodological concerns | Critical methodological concerns in at least one domain |

**Comparison with ROBINS-I**

Table C.3. compares our quality assessment domains with those used in ROBINS-I. We determined that ROBINS-I, while likely a useful tool for some purposes, was unlikely to be a useful metric to gauge the quality or risk of bias of our included studies for several reasons. First, despite its stated aim of providing risk of bias assessment for non-randomized studies of interventions, the way the ROBINS-I tool is constructed appears to be tailored particularly for individual-level cohort designs, whereby individuals receive different interventions and then are followed over time. The detailed guidance for ROBINS-I gives examples of the types of relevant study designs as “cohort studies, case-control studies, controlled before-and-after studies, interrupted-time-series studies and controlled trials in which intervention groups are allocated using a method that falls short of full randomization (sometimes called “quasi-randomized” studies)” (Sterne et al., 2016, p. 3); this may be an issue of disciplinary terminology, but the types of study designs most commonly used to evaluate the effects of state gun policies (e.g., difference-in-differences, synthetic control method, comparative time series) are not referenced.

Perhaps because of this, the way ROBINS-I provides guidance on bias due to confounding is such as to render all studies that would be included in this review as serious or critical risk of bias (e.g., see the Yakubovich et al. [2020] review of self-defense laws which rates every included study as critical or “no information” on the confounding domain of ROBINS-I). The ROBINS-I detailed guidance about this domain also fails to recognize that studies in this field often use quasi-experimental methods whereby the identifying assumptions requisite for interpreting estimated policy effects as causal does not necessitate regression-based adjustments for all baseline or time-varying confounders (which seems to be the focus of the ROBINS-I guidance for judging this domain).

Additionally, ROBINS-I uses some criteria that are rarely relevant for policy evaluation studies like those we include in this review (e.g., selection bias, as defined by ROBINS-I, would almost always receive a “low” rating given its lack of applicability). Answering these would be challenging and time-consuming with little benefit to actually gauging the methodological quality of the studies we review.

There are other domains (e.g., reporting bias) that are difficult to judge given that pre-analysis plans are not conventionally used in this field and thus it would be entirely up to the rater’s judgment regarding whether certain analyses were conducted but not presented in ways likely to misrepresent findings. Particularly because different disciplines and different journals have different expectations about how extensive the number of results and robustness checks should be, it is challenging to fairly and accurately rate these domains in our context.

Finally, there are numerous statistical issues (e.g., number of estimated parameters, distributional assumptions) that have been shown to matter for producing accurate effect estimates and measures of uncertainty, none of which are incorporated into the ROBINS-I tool (Schell et al., 2018, 2022).

Broader issues with evaluator burden and low interrater reliability for ROBINS-I and other assessment tools for nonrandomized studies have been noted elsewhere (Jeyaraman et al., 2020; Minozzi et al., 2019).

**Table C.3 Comparison of domains with Cochrane ROBINS-I**

| ROBINS-I domain | ROBINS-I definition/notes | Our domain | Our definition or reason for NOT including |
| --- | --- | --- | --- |
| Bias due to confounding | The extent to which baseline or time-varying confounding are likely to produce bias in estimated effects | Threats to causal identification | The extent to which a study design is unlikely to produce unbiased estimates of the causal effects of the policies of interest (i.e., a pre-post & comparison group approach is used, but the estimated policy effect should not be interpreted as causal due to violation of the model’s identifying assumptions).  *Note*: This bears some similarities to the ROBINS-I domain but has broader consideration of quasi-experimental methods whereby confounding is not solely addressed through regression-based adjustment. Thus, methods such as difference-in-differences, synthetic control methods, and instrumental variables carry different assumptions and require different judgments than those that are focused on in the ROBINS-I tool. |
| Bias of selection of participants into the study | Selection bias occurs when some eligible participants, or the initial follow up time of some participants, or some outcome events, are excluded in a way that leads to the association between intervention and outcome differing from the association that would have been observed in the target trial | N/A | This does not seem applicable to our type of policy evaluation studies of interest. To the extent that selection bias is a concern in our context, it is covered under the “confounding” domain above. |
| Bias in classification of interventions | Bias may be introduced if there is differential misclassification---when misclassifications of intervention status is related to subsequent outcome or to the risk of the outcome---of intervention status. It is important that, wherever possible, interventions are defined and categorized without knowledge of subsequent outcomes. | Policy classification and coding | With policy evaluation, there are concerns with misclassification but identifying “misclassification” is not straightforward as researchers may rightfully make different decisions about how to group policies depending on the theory guiding their analysis. Thus, we only note this issue for studies that have been subsequently shown to have had serious errors in policy coding that are likely to render their results invalid. |
| Bias due to deviations from intended interventions | When there are systematic differences between the care provided to experimental intervention and comparator groups, beyond the assigned interventions. These differences reflect additional aspects of care, or intended aspects of care that were not delivered. Co-interventions are a potentially important source of bias. | Small number of treated units | Again, in our context this is mostly going to be picked up with the “confounding” domain above. However, with a small number of treated units, there is a much higher risk that something else, such as another policy, occurred coincident with the policy of interest (or subsequent to) in ways that are not easy to address. |
| Bias due to missing data | Differentials in missing data between intervention groups are key, along with the reasons for data being missing. If (i) the proportion of missing data and (ii) the reasons for missing data are similar across intervention groups, then there would typically be only limited bias in the effect estimate, so that risk of bias would be considered low or moderate | Missing data | For almost all of our outcomes, it is difficult to think of a reason missingness might relate to the policy of study. Exceptions are missingness that is a function of the value of the outcome of interest, as the policy effect on the outcome values/levels would then relate to missingness. |
| Bias in measurement of outcomes (misclassification or measurement error) | Bias may be introduced if outcomes are misclassified or measured with error. Differential measurement error is measurement error related intervention status. It will bias the intervention-outcome relationship. This is often referred to as detection bias. | N/A | As with missing data, for almost all included outcomes, it is difficult to think of a reason that outcome measurement would be related to the policy of interest. There are two exceptions to this:   - Justifiable homicide as an outcome for stand-your-ground laws. - Background checks as an outcome for background check policies   We will note these issues in the text but we will not rate this particular domain across all studies. |
| Bias in selection of the reported result | Selective outcome reporting occurs when the effect estimate for an outcome measurement was selected from among analyses of multiple outcome measurements for the outcome domain. Selective analysis reporting occurs when results are selected from intervention effects estimated in multiple ways. Selection of a subgroup from a larger cohort may also provide evidence of selective reporting. | N/A | For low or moderate risk of bias, the ROBINS-I tool here requires a pre-analysis plan, which has just not been the disciplinary standard for these types of policy evaluation studies. Furthermore, there are huge disciplinary and journal differences in the extent to which certain results are expected to be presented and thus it is difficult for us as readers to make judgments calls about which outcomes, analyses, or subgroups may have been evaluated but not shown for selective reporting reasons. |
| N/A |  | Violation of model assumptions & inferential statistics concerns | This domain reflects the potential for fundamental problems with statistical approaches commonly used in the field, which commonly rely on assumptions about independence of residuals, normality, etc. Violating these assumptions often results in inaccurate estimates of uncertainty and, in some cases, can produce biased effect estimates (Schell et al., 2018). |
| N/A |  | Potential for model overfitting | This domain reflects potential issues that arise in overfit models. Including a rich set of covariates can help address concerns with omitted variable bias, but there is a trade-off in that estimating too many parameters relative to the sample size (or relevant to the number of observed events) runs the risk of model overfit, thus potentially reducing the accuracy of the effect estimates and inferential statistics (Peduzzi et al., 1995; Riley et al., 2019). While this will most commonly lead to inflated estimates of variance, in the extreme this can produce severe bias and inflated Type I error. |

**Appendix D: Details on Included Studies and Quality Assessment Results**

Table D.1 lists the included studies, data sources used in the analyses, and the methods used to assess differences in policy effects by race or ethnicity.

To convert estimates from log-link or log-linear models into effect sizes expressed as incidence rate differences (rather than rate ratios), we assumed independence of the estimates across racial groups and converted relative effects to rate differences using an estimate of the mean outcome rate. Mean outcome rates used, and the source underlying the estimates, are provided in Table D.2. Table D.2 also provides details on effect sizes and 95% confidence intervals (CIs) in relative rate terms---the estimates provided within the studies themselves---as well as in rate difference terms. The point estimate for the per capita rate difference (RD) and its upper and lower 95% CIs (UCI and LCI, respectively) are calculated by the following:

$$RD^{i}=\left( IRR^{i}-1 \right)*(mean^{i})$$

$$RD_{LCI}^{i}=\left( IRR_{LCI}^{i}-1 \right)*(mean^{i})$$

$$RD_{UCI}^{i}=\left( IRR_{UCI}^{i}-1 \right)*\left( mean^{i} \right)$$

$for i=w \left( white \right) or nw (nonwhite)$

Table D.2 also indicates whether incidence rate ratios (IRRs) or rate differences (RDs) significantly differed across racial/ethnic subgroups based on the following formulas (unless significance tests were provided in the paper, in which case we used those results).

For IRRs, significance of differences was assessed based on the difference in log(IRRs) as the p-value associated with the following test statistic based on a standard normal distribution:

$$(ln(IRR^{w})-ln(IRR^{nw}))/\sqrt{\left( \frac{\ln\left( {IRR}_{UCI}^{w} \right)-\ln\left( {IRR}_{LCI}^{w} \right)}{2*1.96} \right)^{2}+\left( \frac{\ln\left( {IRR}_{UCI}^{nw} \right)-\ln\left( {IRR}_{LCI}^{nw} \right)}{2*1.96} \right)^{2}}$$

For RDs, significance of differences was assessed based on the difference in RDs as the p-value associated with the following test statistic based on a standard normal distribution:

$$(RD^{w}-RD^{nw})/\sqrt{\left( \frac{{RD}_{UCI}^{w}-{RD}_{LCI}^{w}}{2*1.96} \right)^{2}+\left( \frac{{RD}_{UCI}^{nw}-{RD}_{LCI}^{nw}}{2*1.96} \right)^{2}}$$

Table D.3 presents the quality assessment for each included study estimate (with quality assessed at the study-outcome-policy level). Details for these domains are provided in Tables C.1 and C.2. Based on our quality assessment, three effect comparisons from two studies (Anderson, Sabia, & Tekin, 2021; Degli Esposti et al., 2022) were rated as high methodological quality. Twenty-two effect comparisons across four studies (Anderson et al., 2021; Hepburn et al., 2004; Knopov et al., 2019; Wallin et al., 2022) were rated as having minor methodological concerns. Among these, all were designated as having minor concerns related to limited discussion of or evidence to support the approaches’ identifying assumptions; one study (Knopov et al., 2019) also was flagged for minor concerns due to missing outcome data, and one study (Wallin et al., 2022) did not sufficiently report the number of treated units or policy transitions used in identifying effects. Twenty-four effect comparisons across ten studies (Edwards et al., 2018; Hepburn et al., 2004; Knopov et al. 2019; Lott and Mustard, 1997; McClellan and Tekin, 2017; Olson and Maltz, 2001; Rubin and Dezhbakhsh, 2003; Dalafave, 2021; D’Alession et al., 2022; Pear et al., 2022; Wallin et al., 2022) were rated as having serious methodological concerns. Among these, seven effect comparisons had a small number of treated units with sufficient pre-policy and post-policy data; these were often analyses of relatively recent or rare laws, such as permit-to-purchase requirements (Knopov et al., 2019; Wallin et al., 2022) or permitless carry laws (Knopov et al., 2019). Eight effect comparisons were based on analyses that did not account for potential problems due to systematically missing data. Ten effect comparisons were rated as having serious concerns that the identifying assumptions of the model were met, most commonly because the effects were on policies that were primarily included in models as ancillary covariates rather than the focus of the causal effect estimation. Four effect comparisons were rated as having serious concerns due to violations of statistical assumptions (i.e., not accounting for serial correlation in the data). Finally, we rated one effect comparison as having serious methodological concerns because the outcome data used to identify state policy effects (and analyzed at the state level) are explicitly noted by the data producer as not being appropriate for state-level analysis.

We rated two studies (Rubin and Dezhbakhsh, 2003; Rochford et al., 2022), contributing five effect comparisons, as subject to critical methodological concerns. Rubin and Dezhbakhsh (2003)’s analysis of the role of race in moderating the effects of concealed carry laws is intended to be strictly correlational and not reflective of causal relationships, and their moderation analysis does not adjust the standard errors or other forms of inference to account for the first-stage analysis that generates model-based predictions of the effects of shall-issue law adoption. Rochford et al. (2022) analyze the relationship between domestic violence prohibition policies and intimate partner homicides using a limited timeframe (2013-2016) and controlling only for national trends and a limited set of time-varying state covariates, several of which are plausibly endogenous to the policies (e.g., 2019 violent crime rate). This, combined with fewer than three states transitioning laws during the study period, leads to critical concerns with bias in the effect estimates, which should not be taken as causal and instead likely reflect differences in intimate partner homicide rates by race that are correlated with states that do versus do not have firearm prohibition policies addressing dating partners.

**Table D.1. Details of Included Study Data and Methods**

| Study | Outcome data source(s) | Outcome(s) | Methods | Method for analyzing effects by race | Test for significant difference? |
| --- | --- | --- | --- | --- | --- |
| Anderson et al. | UCR SHR | Homicide rate (juvenile offender) | Poisson | Stratified | No |
|  |  |  |  |  |  |
| Dalafave | CDC WONDER | Homicide rate | Linear model with logged outcome | Stratified | No |
|  |  | Firearm homicide rate | Linear model with logged outcome | Stratified | No |
|  |  | Non-firearm homicide rate | Linear model with logged outcome | Stratified | No |
|  |  | Suicide rate | Linear model with logged outcome | Stratified | No |
|  |  | Firearm suicide rate | Linear model with logged outcome | Stratified | No |
|  |  | Non-firearm suicide rate | Linear model with logged outcome | Stratified | No |
|  |  |  |  |  |  |
| Edwards et al. | NCHS MCOD | Firearm suicide rate | Linear model with logged outcome | Stratified | No |
|  |  |  |  |  |  |
| Hepburn et al. | NCHS MCOD | Homicide rate | Negative binomial | Overall & subgroup analysis | No |
|  |  |  |  |  |  |
| Kaufman et al. | CDC WONDER | Firearm homicide rate | Poisson | Stratified | No |
|  |  |  |  |  |  |
| Knopov et al. | CDC WISQRS | Homicide rate, age-adjusted | Linear model with logged outcome | 1) model policy interacted with race  2) for significant interaction terms, conduct stratified analysis | Yes |
|  |  |  |  |  |  |
| Lott & Mustard | UCR SHR | % of murders with victim of certain race or ethnicity | Linear model with logged outcome | Define dependent variable with respect to racial composition | Implicit |
|  |  |  |  |  |  |
| McClellan & Tekin | NCHS MCOD | Firearm homicide rate | Poisson* | Stratified | No |
|  | UCR SHR | Justifiable homicide rate | Poisson* | Stratified | No |
|  | SEDDs & SIDs | Intentional firearm injury hospitalization/ED rate | Linear with logged outcome | Stratified | No |
|  | NIS | Intentional firearm injury hospitalization rate | Linear with logged outcome | Stratified | No |
|  |  |  |  |  |  |
|  |  |  |  |  |  |
| Olson & Maltz | UCR SHR | % of murders with victim of certain race or ethnicity | Linear model with logged outcome | Define dependent variable with respect to racial composition | Implicit |
|  |  |  |  |  |  |
| Rubin & Dzhbakhsh | UCR SHR | Predicted change in violent crime | Linear | Predictors include racial composition of county | Implicit |
|  |  |  |  |  |  |
| D’Alessio et al. | NIBRS | % crime incidents involving a firearm | Linear | Interact policy variables with: % of firearm crime incidents with black offender | Implicit |
|  |  |  |  |  |  |
| Degli Esposti | NCHS MCOD | Homicide rate | Quasi-Poisson | Stratified | Yes |
|  |  | Firearm homicide rate | Quasi-Poisson | Stratified | Yes |
|  |  |  |  |  |  |
| Pear et al. | CA Department of Public Health; CA Office of Statewide Health Planning and Development | Firearm assault rate | Synthetic control method (linear) | Stratified | No |
|  |  | Intentional firearm self-injury rate | Synthetic control method (linear) | Overall & subgroup analysis | No |
|  |  |  |  |  |  |
| Rochford | NVDRS | IPH (unmarried victims) | Negative binomial | Stratified | No |
|  |  |  |  |  |  |
| Wallin et al. | UCR SHR | IPH rate | Negative binomial | Stratified | No |
|  |  | Firearm IPH rate | Negative binomial | Stratified | No |

NOTES: NCHS = National Center for Health Statistics. UCR = Uniform Crime Reports.

**Table D.2. Details of Included Study Outcomes and Findings for Policy Effects**

| Study | Policy | Outcome | Race | IRR (95% CI) | p-val ratio | Mean estimate | Mean source | RD (95% CI) | p-val diff |
| --- | --- | --- | --- | --- | --- | --- | --- | --- | --- |
| **Policies regulating legal uses, carrying, and storage of firearms** | | | | | | | | | |
| Knopov | Stand-your-ground | Total homicide | White | 1.03 (0.97, 1.09) | ns^a^ | 3.96 | CDC WONDER | 0.12 (-0.13, 0.37) | 0.46 |
|  |  |  | Nonwhite | 1.03 (0.97, 1.09) |  | 23.81 | CDC WONDER | 0.69 (-0.76, 2.21) |  |
| Degli Esposti | Stand-your-ground | Total homicide | White | 1.10 (1.05, 1.15) | 0.25 | 3.96 | Table (before law) | 0.40 (0.20, 0.59) | 0.34 |
|  |  |  | Nonwhite | 1.05 (1.00, 1.11) |  | 17.40 | Table (before law) | 0.87 (0.00, 1.91) |  |
|  |  | Firearm homicide | White | 1.10 (1.04, 1.17) | 0.20 | 2.40 | Table (before law) | 0.24 (0.10, 0.41) | 0.57 |
|  |  |  | Nonwhite | 1.04 (0.97, 1.11) |  | 12.36 | Table (before law) | 0.49 (-0.37, 1.36) |  |
| McClellan | Stand-your-ground | Firearm homicide | White | 1.22 (1.11, 1.34) | 0.02 | 2.15 | CDC WONDER | 0.48 (0.25, 0.73) | 0.55 |
|  |  |  | Nonwhite | 0.98 (0.83, 1.15) |  | 16.21 | CDC WONDER | -0.34 (-2.75, 2.50) |  |
|  |  | Non-justifiable homicide | White | 1.17 (1.01, 1.34) | 0.25 | 1.72 | SHR & NCHS | 0.29 (0.02, 0.60) | 0.78 |
|  |  |  | Nonwhite | 1.04 (0.92, 1.19) |  | 13.39 | SHR & NCHS | 0.55 (-1.15, 2.47) |  |
|  |  | Justifiable homicide | White | 2.53 (1.67, 3.83) | 0.001 | 0.03 | SHR & NCHS | 0.05 (0.02, 0.09) | 0.06 |
|  |  |  | Nonwhite | 0.73 (0.42, 1.29) |  | 0.27 | SHR & NCHS | -0.07 (-0.16, 0.08) |  |
|  |  | Firearm assault hospitalizations | White | 1.43 (0.66, 3.11) | 0.91 | NI |  |  |  |
|  |  |  | Nonwhite | 1.35 (0.63, 2.88) |  | NI |  |  |  |
|  | Stand-your-ground (FL) | Firearm assault ED/hospitalizations | White | 1.12 (1.00, 1.26) | 0.11 | NI |  |  |  |
|  |  |  | Nonwhite | 1.02 (0.97, 1.06) |  | NI |  |  |  |
| Knopov | Shall-issue law | Total homicide | White | 1.06 (1.04, 1.11) | ns^a^ | 3.96 | CDC WONDER | 0.23 (0.16, 0.45) | 0.01 |
|  |  |  | Nonwhite | 1.06 (1.04, 1.11) |  | 23.81 | CDC WONDER | 1.36 (0.95, 2.69) |  |
|  | Permitless carry | Total homicide | White | 0.94 (0.86, 1.02) | ns^a^ | 3.96 | CDC WONDER | -0.25 (-0.57, 0.09) | 0.22 |
|  |  |  | Nonwhite | 0.94 (0.86, 1.02) |  | 23.81 | CDC WONDER | -1.50 (-3.40, 0.57) |  |
| Hepburn | Shall-issue law | Total homicide | White^b^ | 1.03 (0.94, 1.11) | N/A |  |  |  |  |
|  |  |  | Nonwhite | N/A |  |  |  |  |  |
| Anderson | CAP Law | Firearm homicide | White | 0.77 (0.62, 0.95) | 0.30 | 0.66 | Table 6 and SHR | -0.15 (-0.25, -0.03) | 0.30 |
|  |  |  | Nonwhite | 0.89 (0.75, 1.05) |  | 4.64 | Table 6 and SHR | -0.40 (-1.17, 0.23) |  |
| **Policies regulating firearm sales and transfers** | | | | | | | | | |
| Edwards | Waiting period | Firearm suicide | White | 0.98 (0.96, 1.00) | 0.38 | 7.19 | CDC WONDER | -0.16 (-0.32, 0.01) | 0.42 |
|  |  |  | Nonwhite | 0.94 (0.87, 1.02) |  | 6.28 | CDC WONDER | -0.37 (-0.85, 0.15) |  |
| Hepburn | Waiting period | Total homicide | White^b^ | 0.99 (0.89, 1.11) | N/A |  |  |  |  |
|  |  |  | Nonwhite | N/A |  |  |  |  |  |
| Kaufman | UBC | Firearm homicide | White | 0.93 (0.73, 1.20) | 0.35 | 1.80 | Table 2 | -0.13 (-0.49, 0.36) | 0.004 |
|  |  |  | Nonwhite | 0.81 (0.70, 0.94) |  | 15.60 | Table 2 | -2.96 (-4.68, -0.94) |  |
| Knopov | UBC | Total homicide | White | 0.88 (0.81, 0.97) | ns^a^ | 3.96 | CDC WONDER | -0.44 (-0.75, -0.11) | 0.03 |
|  |  |  | Nonwhite | 0.88 (0.81, 0.97) |  | 23.81 | CDC WONDER | -2.67 (-4.52, -0.67) |  |
| Wallin | UBC | IPH | White | 1.05 (0.90, 1.24) | 0.71 | 0.75 | Figure 1a (est) | 0.04 (-0.08, 0.18) | 0.25 |
|  |  |  | Nonwhite | 1.09 (0.97, 1.23) |  | 3.50 | Figure 1a (est) | 0.32 (-0.11, 0.81) |  |
|  |  | Firearm IPH | White | 1.18 (0.95, 1.46) | 0.54 | 0.50 | Figure 1b (est) | 0.09 (-0.03, 0.23) | 0.74 |
|  |  |  | Nonwhite | 1.08 (0.90, 1.29) |  | 2.00 | Figure 1b (est) | 0.16 (-0.20, 0.58) |  |
| Hepburn | UBC | Total homicide | White^b^ | 0.97 (0.88, 1.06) | N/A |  |  |  |  |
|  |  |  | Nonwhite | N/A |  |  |  |  |  |
| Wallin | POC Background check | IPH | White | 0.97 (0.89, 1.06) | 0.88 | 0.75 | Figure 1a (est) | -0.02 (-0.08, 0.05) | 0.78 |
|  |  |  | Nonwhite | 0.98 (0.89, 1.08) |  | 3.50 | Figure 1a (est) | -0.07 (-0.39, 0.28) |  |
|  |  | Firearm IPH | White | 0.99 (0.88, 1.11) | 0.47 | 0.50 | Figure 1b (est) | -0.01 (-0.06, 0.06) | 0.26 |
|  |  |  | Nonwhite | 0.93 (0.82, 1.05) |  | 2.00 | Figure 1b (est) | -0.14 (-0.36, 0.10) |  |
| Knopov | Permit-to-purchase | Total homicide | White | 0.89 (0.80, 0.99) | ns | 3.96 | CDC WONDER | -0.45 (-0.79, -0.06) | 0.05 |
|  |  |  | Nonwhite | 0.89 (0.80, 0.99) |  | 23.81 | CDC WONDER | -2.69 (-4.76, -0.36) |  |
| Wallin | Permit-to-purchase | IPH | White | 1.05 (0.88, 1.25) | 0.91 | 0.75 | Figure 1a (est) | 0.04 (-0.04, 0.19) | 0.90 |
|  |  |  | Nonwhite | 1.03 (0.77, 1.36) |  | 3.50 | Figure 1a (est) | 0.11 (-0.81, 1.26) |  |
|  |  | Firearm IPH | White | 1.07 (0.92, 1.25) | 0.78 | 0.50 | Figure 1b (est) | 0.03 (-0.04, 0.12) | 0.68 |
|  |  |  | Nonwhite | 1.16 (0.66, 2.01) |  | 2.00 | Figure 1b (est) | 0.32 (-0.68, 2.02) |  |
| **Policies regulating who can possess or purchase firearms** | | | | | | | | | |
| Knopov | DVRO+VM+Surr | Total homicide | White | 0.97 (0.92, 1.02) | 0.03 | 3.96 | CDC WONDER | -0.13 (-0.33, 0.09) | 0.001 |
|  |  |  | Nonwhite | 0.87 (0.81, 0.94) |  | 23.81 | CDC WONDER | -3.05 (-4.62, -1.33) |  |
| Rochford | Extend DVRO and DVM prohib to dating partners | IPH (unmarried)^c^ | White | 0.72 (0.57, 0.92) | 0.003 | 1183.00 | Table 2, no closure | -328 (-509, -95) | 0.001 |
|  |  |  | Nonwhite | 1.28 (0.96, 1.70) |  | 629.00 | Table 2, no closure | 177 (-25, 440) |  |
|  | Extend DVRO or DVM prohib to dating partners | IPH (unmarried)^c^ | White | 0.57 (0.35, 0.94) | 0.04 | 1183.00 | Table 2, no closure | -500 (-769, -71) | 0.04 |
|  |  |  | Nonwhite | 1.34 (0.71, 2.52) |  | 629.00 | Table 2, no closure | 214 (-182, 956) |  |
|  | Extend DVM prohibition only to dating partners | IPH (unmarried)^c^ | White | 0.57 (0.35, 0.94) | 0.04 | 1183.00 | Table 2, no closure | -500 (-769, -71) | 0.04 |
|  |  |  | Nonwhite | 1.34 (0.71, 2.52) |  | 629.00 | Table 2, no closure | 214 (-182, 956) |  |
|  | Extend DVRO prohibition only to dating partners | IPH (unmarried)^c^ | White | 0.55 (0.39, 0.78) | 0.000 | 1183.00 | Table 2, no closure | -525 (-722, -260) | 0.000 |
|  |  |  | Nonwhite | 1.60 (1.10, 2.35) |  | 629.00 | Table 2, no closure | 383 (63, 849) |  |
| Wallin | State DVRO | IPH | White | 0.90 (0.83, 0.98) | 0.15 | 0.75 | Figure 1a (est) | -0.08 (-0.13, -0.02) | 0.65 |
|  |  |  | Nonwhite | 1.01 (0.88, 1.15) |  | 3.50 | Figure 1a (est) | 0.04 (-0.42, 0.53) |  |
|  |  | Firearm IPH | White | 0.89 (0.80, 0.99) | 0.36 | 0.50 | Figure 1b (est) | -0.06 (-0.10, -0.01) | 0.93 |
|  |  |  | Nonwhite | 0.98 (0.82, 1.17) |  | 2.00 | Figure 1b (est) | -0.04 (-0.36, 0.34) |  |
|  | State DVM | IPH | White | 0.99 (0.85, 1.16) | 0.92 | 0.75 | Figure 1a (est) | -0.01 (-0.11, 0.12) | 0.98 |
|  |  |  | Nonwhite | 1.00 (0.88, 1.14) |  | 3.50 | Figure 1a (est) | 0.00 (-0.42, 0.49) |  |
|  |  | Firearm IPH | White | 1.04 (0.87, 1.24) | 0.87 | 0.50 | Figure 1b (est) | 0.02 (-0.07, 0.12) | 0.56 |
|  |  |  | Nonwhite | 1.06 (0.91, 1.23) |  | 2.00 | Figure 1b (est) | 0.12 (-0.18, 0.46) |  |
|  | VM prohibition | IPH | White | 0.77 (0.63, 0.95) | 0.07 | 0.75 | Figure 1a (est) | -0.17 (-0.28, -0.04) | 0.79 |
|  |  |  | Nonwhite | 0.97 (0.84, 1.12) |  | 3.50 | Figure 1a (est) | -0.11 (-0.56, 0.42) |  |
|  |  | Firearm IPH | White | 0.72 (0.53, 0.98) | 0.06 | 0.50 | Figure 1b (est) | -0.14 (-0.24, -0.01) | 0.38 |
|  |  |  | Nonwhite | 1.05 (0.82, 1.34) |  | 2.00 | Figure 1b (est) | 0.10 (-0.36, 0.68) |  |
|  | Stalking misdemeanor prohibitions | IPH | White | 1.2 (1.04, 1.38) | 0.13 | 0.75 | Figure 1a (est) | 0.15 (0.03, 0.28) | 0.86 |
|  |  |  | Nonwhite | 1.03 (0.90, 1.18) |  | 3.50 | Figure 1a (est) | 0.11 (-0.35, 0.63) |  |
|  |  | Firearm IPH | White | 1.15 (0.95, 1.40) | 0.26 | 0.50 | Figure 1b (est) | 0.08 (-0.03, 0.20) | 0.56 |
|  |  |  | Nonwhite | 0.97 (0.78, 1.22) |  | 2.00 | Figure 1b (est) | -0.06 (-0.44, 0.44) |  |
|  | Stalking felony | IPH | White | 1.00 (0.90, 1.12) | 0.64 | 0.75 | Figure 1a (est) | 0.00 (-0.08, 0.09) | 0.54 |
|  |  |  | Nonwhite | 1.04 (0.92, 1.17) |  | 3.50 | Figure 1a (est) | 0.14 (-0.28, 0.59) |  |
|  |  | Firearm IPH | White | 0.97 (0.81, 1.16) | 0.94 | 0.50 | Figure 1b (est) | -0.02 (-0.10, 0.08) | 0.72 |
|  |  |  | Nonwhite | 0.96 (0.80, 1.14) |  | 2.00 | Figure 1b (est) | -0.08 (-0.40, 0.28) |  |
|  | Federal DVM | IPH | White | 0.93 (0.86, 1.01) | 0.75 | 0.75 | Figure 1a (est) | -0.05 (-0.10, 0.08) | 0.48 |
|  |  |  | Nonwhite | 0.95 (0.86, 1.05) |  | 3.50 | Figure 1a (est) | -0.18 (-0.49, 0.17) |  |
|  |  | Firearm IPH | White | 0.93 (0.84, 1.03) | 0.47 | 0.50 | Figure 1b (est) | -0.04 (-0.08, 0.02) | 0.10 |
|  |  |  | Nonwhite | 0.87 (0.75, 1.01) |  | 2.00 | Figure 1b (est) | -0.26 (-0.50, 0.02) |  |
|  | Surrender | IPH | White | 0.98 (0.86, 1.13) | 0.35 | 0.75 | Figure 1a (est) | -0.02 (-0.10, 0.10) | 0.27 |
|  |  |  | Nonwhite | 1.07 (0.95, 1.21) |  | 3.50 | Figure 1a (est) | 0.24 (-0.18, 0.73) |  |
|  |  | Firearm IPH | White | 0.98 (0.82, 1.16) | 0.69 | 0.50 | Figure 1b (est) | -0.01 (-0.09, 0.08) | 0.71 |
|  |  |  | Nonwhite | 1.03 (0.86, 1.22) |  | 2.00 | Figure 1b (est) | 0.06 (-0.28, 0.44) |  |
| Dalafave | ERPO law | Total suicide | White | 0.96 (0.93, 0.98) | 0.08 | 14.73 | Table 1: No red flag law | -0.55 (-0.91, -0.18) | 0.12 |
|  |  |  | Nonwhite | 0.87 (0.78, 0.97) |  | 11.31 | Table 1: No red flag law | -1.44 (-2.45, -0.31) |  |
|  |  | Firearm suicide | White | 0.93 (0.89, 0.96) | 0.84 | 8.59 | Table 1: No red flag law | -0.58 (-0.89, -0.26) | 0.67 |
|  |  |  | Nonwhite | 0.91 (0.78, 1.07) |  | 4.90 | Table 1: No red flag law | -0.41 (-1.08, 0.38) |  |
|  |  | Nonfirearm suicide | White | 1.00 (0.97, 1.04) | 0.10 | 6.23 | Table 1: No red flag law | 0.03 (-0.19, 0.26) | 0.08 |
|  |  |  | Nonwhite | 0.90 (0.79, 1.01) |  | 6.47 | Table 1: No red flag law | -0.64 (-1.32, 0.13) |  |
|  |  | Total homicide | White | 1.04 (0.91, 1.19) | 0.21 | 3.82 | Table 1: No red flag law | 0.17 (-0.33, 0.74) | 0.20 |
|  |  |  | Nonwhite | 0.92 (0.80, 1.05) |  | 19.79 | Table 1: No red flag law | -1.50 (-3.85, 1.19) |  |
|  |  | Firearm homicide | White | 1.00 (0.82, 1.22) | 0.46 | 2.34 | Table 1: No red flag law | -0.01 (-0.42, 0.51) | 0.33 |
|  |  |  | Nonwhite | 1.11 (0.91, 1.34) |  | 14.18 | Table 1: No red flag law | 1.51 (-1.23, 4.83) |  |
|  |  | Nonfirearm homicide | White | 1.11 (1.02, 1.21) | 0.16 | 1.53 | Table 1: No red flag law | 0.18 (0.04, 0.33) | 0.59 |
|  |  |  | Nonwhite | 0.99 (0.87, 1.14) |  | 5.59 | Table 1: No red flag law | -0.03 (-0.74, 0.77) |  |
| Pear | ERPO in San Diego | Firearm assault | White | 0.76 (0.16, 1.71) | 0.99 |  | Rate diff provided | -0.66 (-1.86, 0.54) | 0.36 |
|  |  |  | Nonwhite | 0.72 (0.00, 9.44) |  |  | Rate diff provided | -3.37 (-8.99, 2.24) |  |
|  |  | Intentional firearm self-harm | White | 0.95 (0.30, 1.46) | N/A |  |  |  |  |
|  |  |  | Nonwhite | N/A |  |  |  |  |  |

NOTES:

Studies of high methodological criteria are shaded white, studies with minor methodological concerns are shaded light gray, studies with serious methodological concerns are shaded medium gray, and studies with critical methodological concerns are shaded dark gray. Quality criteria assessment ratings are provided in Table D.3.

Table does not include results from studies that analyzed change in composition of crimes (D’Alessio et al., 2022; Lott and Mustard, 1997; Olson and Maltz, 2001), nor the moderation analysis of Rubin & Dezhbakhsh (2003).

UBC = universal background check. PTP = permit-to-purchase. CAP=child access prevention. IRR = incidence rate ratio. RD = rate difference. CI = confidence interval. NI = not identified.

^a^The approach by Knopov et al. first conducted a pooled analyses in which they controlled for a main policy effect and an interaction effect (policy*nonwhite). If the interaction effect was not significant (ns), they re-estimate the model without the interaction term, thus treating both racial groups as experiencing the same relative effect size; this is why for policies that showed no significant difference, the IRRs and 95% CIs are the same for both white and nonwhite outcomes shown in this table. In contrast, for policies that had a significant interaction effect, the authors re-estimated the models stratified by race; for these policies, we report the IRRs and 95% CIs from the stratified models presented in the study, while calculating p-values based on the stratified results (assuming independence).

^b^Estimate in the column for white is restricted to white males ages 35+ in the Hepburn et al. study.

^c^Estimates in Rochford et al. are in counts (number of incidents) rather than rates.

**Table D.3. Quality assessment of included study estimates**

| **Study** | **Outcome** | **Policy** | **Threat to Identification** | **Sample Size/Deviation** | **Policy Classification** | **Missing data** | **Potential model overfit** | **Statistical assumptions** | **Other** | **OVERALL** |
| --- | --- | --- | --- | --- | --- | --- | --- | --- | --- | --- |
| Anderson et al. | Firearm murder | Child access prevention law | M | 0 | 0 | 0 | M | 0 | 0 | M |
| Edwards et al. | Firearm suicide | Waiting period requirement | M | 0 | 0 | S | 0 | 0 | 0 | S |
| Hepburn et al. | Total homicide | Shall issue law | M | 0 | 0 | 0 | 0 | 0 | 0 | M |
| Hepburn et al. | Total homicide | Universal background check for handguns | S | M | 0 | 0 | 0 | 0 | 0 | S |
| Hepburn et al. | Total homicide | Waiting period requirement | S | M | 0 | 0 | 0 | 0 | 0 | S |
| Kaufman et al. | Firearm homicide | Universal background check for handguns | M | 0 | 0 | S | M | 0 | 0 | S |
| Knopov et al. | Total homicide | Universal background check | M | 0 | 0 | M | 0 | 0 | 0 | M |
| Knopov et al. | Total homicide | Shall issue law | M | 0 | 0 | M | 0 | 0 | 0 | M |
| Knopov et al. | Total homicide | Permitless carry law | M | S | 0 | M | 0 | 0 | 0 | S |
| Knopov et al. | Total homicide | Permit-to-purchase requirement | M | S | 0 | M | 0 | 0 | 0 | S |
| Knopov et al. | Total homicide | Stand your ground law | M | 0 | 0 | M | 0 | 0 | 0 | M |
| Knopov et al. | Total homicide | DV or VM handgun prohibition + surrender | M | 0 | 0 | M | 0 | 0 | 0 | M |
| Lott & Mustard | Murder composition | Shall issue law | M | 0 | 0 | 0 | M | S | 0 | S |
| McClellan & Tekin | Firearm homicide | Stand your ground law | 0 | 0 | 0 | 0 | 0 | 0 | 0 | 0 |
| McClellan & Tekin | Non-justifiable homicide | Stand your ground law | M | 0 | 0 | 0 | C | 0 | 0 | C |
| McClellan & Tekin | Justifiable homicide | Stand your ground law | M | 0 | 0 | 0 | C | 0 | 0 | C |
| McClellan & Tekin | Firearm assault ED/inpatient | Stand your ground law | M | S | 0 | 0 | C | 0 | 0 | C |
| McClellan & Tekin | Firearm assault inpatient | Stand your ground law | M | 0 | 0 | 0 | M | 0 | S | S |
| Olson & Maltz | Murder composition | Shall issue law | M | 0 | 0 | 0 | 0 | S | 0 | S |
| Rubin & Dezhbakhsh | Violent crime | Shall issue law (moderation analysis) | C | 0 | 0 | 0 | 0 | S | 0 | C |
| Dalafave | Total suicide | Extreme risk protection order (ERPO) law | M | 0 | 0 | S | 0 | 0 | 0 | S |
| Dalafave | Firearm suicide | Extreme risk protection order (ERPO) law | M | 0 | 0 | S | 0 | 0 | 0 | S |
| Dalafave | Non-firearm suicide | Extreme risk protection order (ERPO) law | M | 0 | 0 | S | 0 | 0 | 0 | S |
| Dalafave | Total homicide | Extreme risk protection order (ERPO) law | M | 0 | 0 | S | 0 | 0 | 0 | S |
| Dalafave | Firearm homicide | Extreme risk protection order (ERPO) law | M | 0 | 0 | S | 0 | 0 | 0 | S |
| Dalafave | Non-firearm homicide | Extreme risk protection order (ERPO) law | M | 0 | 0 | S | 0 | 0 | 0 | S |
| D’Alessio et al. | Crime composition | Stand your ground law (moderation analysis) | S | 0 | 0 | 0 | 0 | S | 0 | S |
| Degli Esposti et al. | Total homicide | Stand your ground law | 0 | 0 | 0 | 0 | 0 | 0 | 0 | 0 |
| Degli Esposti et al. | Firearm homicide | Stand your ground law | 0 | 0 | 0 | 0 | 0 | 0 | 0 | 0 |
| Pear et al. | Firearm assault | Extreme risk protection order | S | S | 0 | 0 | 0 | 0 | 0 | S |
| Pear et al. | Intentional firearm self-harm | Extreme risk protection order | M | S | 0 | 0 | 0 | 0 | 0 | S |
| Rochford et al. | Intimate partner homicide | Extend DVRO and DVM to dating partners | C | S | 0 | 0 | M | 0 | 0 | C |
| Rochford et al. | Intimate partner homicide | Extend DVRO or DVM to dating partners | C | S | 0 | 0 | M | 0 | 0 | C |
| Rochford et al. | Intimate partner homicide | Extend DVM (only) to dating partners | C | S | 0 | 0 | M | 0 | 0 | C |
| Rochford et al. | Intimate partner homicide | Extend DVRO (only) to dating partners | C | S | 0 | 0 | M | 0 | 0 | C |
| Wallin et al. | Intimate partner homicide | State DVRO prohibition | M | M | 0 | 0 | 0 | 0 | 0 | M |
| Wallin et al. | Firearm intimate partner homicide | State DVRO prohibition | M | M | 0 | 0 | 0 | 0 | 0 | M |
| Wallin et al. | Intimate partner homicide | State DVM prohibition | M | M | 0 | 0 | 0 | 0 | 0 | M |
| Wallin et al. | Firearm intimate partner homicide | State DVM prohibition | M | M | 0 | 0 | 0 | 0 | 0 | M |
| Wallin et al. | Intimate partner homicide | Stalking misdemeanor prohibition | M | M | 0 | 0 | 0 | 0 | 0 | M |
| Wallin et al. | Firearm intimate partner homicide | Stalking misdemeanor prohibition | M | M | 0 | 0 | 0 | 0 | 0 | M |
| Wallin et al. | Intimate partner homicide | Felony stalking law | M | M | 0 | 0 | 0 | 0 | 0 | M |
| Wallin et al. | Firearm intimate partner homicide | Felony stalking law | M | M | 0 | 0 | 0 | 0 | 0 | M |
| Wallin et al. | Intimate partner homicide | Federal DVM prohibition | M | M | 0 | 0 | 0 | 0 | 0 | M |
| Wallin et al. | Firearm intimate partner homicide | Federal DVM prohibition | M | M | 0 | 0 | 0 | 0 | 0 | M |
| Wallin et al. | Intimate partner homicide | Permit-to-purchase requirement | S | S | 0 | 0 | 0 | 0 | 0 | S |
| Wallin et al. | Firearm intimate partner homicide | Permit-to-purchase requirement | S | S | 0 | 0 | 0 | 0 | 0 | S |
| Wallin et al. | Intimate partner homicide | Universal background checks | S | M | 0 | 0 | 0 | 0 | 0 | S |
| Wallin et al. | Firearm intimate partner homicide | Universal background checks | S | M | 0 | 0 | 0 | 0 | 0 | S |
| Wallin et al. | Intimate partner homicide | Point-of-contact background check | S | M | 0 | 0 | 0 | 0 | 0 | S |
| Wallin et al. | Firearm intimate partner homicide | Point-of-contact background check | S | M | 0 | 0 | 0 | 0 | 0 | S |
| Wallin et al. | Intimate partner homicide | Relinquishment law (DV scene) | M | M | 0 | 0 | 0 | 0 | 0 | M |
| Wallin et al. | Firearm intimate partner homicide | Relinquishment law (DV scene) | M | M | 0 | 0 | 0 | 0 | 0 | M |
| Wallin et al. | Intimate partner homicide | DVRO and no relinquishment | M | M | 0 | 0 | 0 | 0 | 0 | M |
| Wallin et al. | Firearm intimate partner homicide | DVRO and no relinquishment | M | M | 0 | 0 | 0 | 0 | 0 | M |
| Wallin et al. | Intimate partner homicide | DVRO and relinquishment | M | M | 0 | 0 | 0 | 0 | 0 | M |
| Wallin et al. | Firearm intimate partner homicide | DVRO and relinquishment | M | M | 0 | 0 | 0 | 0 | 0 | M |

NOTES: 0: no methodological concerns of note. M: minor methodological concerns. S: serious methodological concerns. C: critical methodological concerns. ERPO = extreme risk protection order. DV = domestic violence. DVRO = domestic violence restraining order. DVM = domestic violence misdemeanor.

**Supplemental Materials References**

Humphreys, David K., Antonio Gasparrini, and Douglas J. Wiebe, “Evaluating the Impact of Florida’s ‘Stand Your Ground’ Self-Defense Law on Homicide and Suicide by Firearm: An Interrupted Time Series Study,” JAMA Internal Medicine, Vol. 177, No. 1, 2017, pp. 44–50.

Hünermund, Paul, and Beyers Louw. On the Nuisance of Control Variables in Regression Analysis. Arxiv 2005.10314v4. September 28, 2022. Available at: <https://arxiv.org/abs/2005.10314>.

Jeyaraman, Maya M., Rasheda Rabbani, Leslie Copstein, Reid C. Robson, Nameer Al-Yousif, Michelle Pollock, Jun Xia et al. "Methodologically rigorous risk of bias tools for nonrandomized studies had low reliability and high evaluator burden." Journal of clinical epidemiology 128 (2020): 140-147.

Minozzi, Silvia, Michela Cinquini, Silvia Gianola, Greta Castellini, Chiara Gerardi, and Rita Banzi. "Risk of bias in nonrandomized studies of interventions showed low inter-rater reliability and challenges in its application." Journal of clinical epidemiology 112 (2019): 28-35.

Peduzzi, P., Concato, J., Feinstein, A. R., & Holford, T. R. (1995). Importance of events per independent variable in proportional hazards regression analysis II. Accuracy and precision of regression estimates. Journal of Clinical Epidemiology, 48(12), 1503–1510.

Riley, Richard D., Kym I. E. Snell, Joie Ensor, Danielle L. Burke, Frank E. Harrell, Jr., Karel G. M. Moons, and Gary S. Collins, “Minimum Sample Size for Developing a Multivariable Prediction Model: Part I–Continuous Outcomes,” Statistics in Medicine, Vol. 38, No. 7, 2019, pp. 1262–1275.

Schell, Terry L., Beth Ann Griffin, and Andrew R. Morral. Evaluating methods to estimate the effect of state laws on firearm deaths: A simulation study. RAND Corporation, 2018.

Schell, Terry L., Rosanna Smart, and Andrew R. Morral Suggestions for Estimating the Effects of State Gun Policies: Commentary on Four Methodological Problems in the Current Literature. RAND Corporation, 2022.

Smart, Rosanna, Kelsey O’Halloren, James Murphy, Sierra Smucker, Rupa Jose, Pierrce Holmes, Sangeeta Ahluwalia, Terry L. Schell, and Andrew R. Morral. In progress. Systematic Review of the Effects of Gun Policies in the United States, Fourth Edition (UPDATE). PROSPERO 2023 CRD42023410904 Available from: <https://www.crd.york.ac.uk/prospero/display_record.php?ID=CRD42023410904>

Smart, Rosanna, Andrew R. Morral, Rajeev Ramchand, Amanda Charbonneau, Jhacova Williams, Sierra Smucker, Samantha Cherney, and Lea Xenakis. The science of gun policy: a critical synthesis of research evidence on the effects of gun policies in the United States. RAND Corporation, 2023.

Sterne JAC, Higgins JPT, Elbers RG, Reeves BC and the development group for ROBINS-I. Risk Of Bias In Non-randomized Studies of Interventions (ROBINS-I): detailed guidance, updated 12 October 2016.

Yakubovich, Alexa R., Michelle Degli Esposti, Brittany CL Lange, G. J. Melendez-Torres, Alpa Parmar, Douglas J. Wiebe, and David K. Humphreys. "Effects of laws expanding civilian rights to use deadly force in self-defense on violence and crime: a systematic review." American journal of public health 111, no. 4 (2021): e1-e14.
